# Prediction of Smoking Behavior From Single Nucleotide Polymorphisms With Machine Learning Approaches

**DOI:** 10.3389/fpsyt.2020.00416

**Published:** 2020-05-14

**Authors:** Yi Xu, Liyu Cao, Xinyi Zhao, Yinghao Yao, Qiang Liu, Bin Zhang, Yan Wang, Ying Mao, Yunlong Ma, Jennie Z. Ma, Thomas J. Payne, Ming D. Li, Lanjuan Li

**Affiliations:** ^1^State Key Laboratory for Diagnosis and Treatment of Infectious Diseases, National Clinical Research Center for Infectious Diseases, Collaborative Innovation Center for Diagnosis and Treatment of Infectious Diseases, The First Affiliated Hospital, Zhejiang University School of Medicine, Hangzhou, China; ^2^Department of Public Health Sciences, University of Virginia, Charlottesville, VA, United States; ^3^Department of Otolaryngology and Communicative Sciences, University of Mississippi Medical Center, Jackson, MS, United States; ^4^Research Center for Air Pollution and Health, Zhejiang University, Hangzhou, China

**Keywords:** machine learning, prediction of smoking, single-nucleotide polymorphisms, smoking, feature selection

## Abstract

Smoking is a complex behavior with a heritability as high as 50%. Given such a large genetic contribution, it provides an opportunity to prevent those individuals who are susceptible to smoking dependence from ever starting to smoke by predicting their inherited predisposition with their genomic profiles. Although previous studies have identified many susceptibility variants for smoking, they have limited power to predict smoking behavior. We applied the support vector machine (SVM) and random forest (RF) methods to build prediction models for smoking behavior. We first used 1,431 smokers and 1,503 non-smokers of African origin for model building with a 10-fold cross-validation and then tested the prediction models on an independent dataset consisting of 213 smokers and 224 non-smokers. The SVM model with 500 top single nucleotide polymorphisms (SNPs) selected using logistic regression (p<0.01) as the feature selection method achieved an area under the curve (AUC) of 0.691, 0.721, and 0.720 for the training, test, and independent test samples, respectively. The RF model with 500 top SNPs selected using logistic regression (p<0.01) achieved AUCs of 0.671, 0.665, and 0.667 for the training, test, and independent test samples, respectively. Finally, we used the combined logistic (p<0.01) and LASSO (λ=10^−3^) regression to select features and the SVM algorithm for model building. The SVM model with 500 top SNPs achieved AUCs of 0.756, 0.776, and 0.897 for the training, test, and independent test samples, respectively. We conclude that machine learning methods are promising means to build predictive models for smoking.

## Introduction

Tobacco smoking is one of the most important public health problems throughout the world (1). According to a World Health Organization report, the number of deaths caused by tobacco smoking will reach 10 million worldwide annually by 2020 (2). Without significant efforts to limit tobacco smoking, this number will rise to 8.3 million by 2030 (3). Thus, prevention of smoking initiation has become a critical step in tobacco control (4–7). Stopping individuals susceptible to nicotine dependence from starting to smoke represents an effective way to achieve tobacco control.

Tobacco smoking is a complex and multifactorial behavior determined by both genetic and environmental factors, as well as by gene-by-gene and gene-by-environmental interactions (8, 9). The heritability of various smoking-related phenotypes ranges from 21 to 84% (10–13). Thus, it is feasible to predict an individual's inherited predisposition to smoking on the basis of the genomic profile.

Numerous genetic investigations using various approaches such as genome-wide linkage analysis, candidate gene-based association, and genome-wide association studies (GWAS) have revealed a number of variants that make persons susceptible to tobacco smoking (14–18). However, these variants explain only a small proportion of the known heritability, and their individual contribution to smoking behavior becomes almost negligible, which leads to difficulty in predicting the phenotype from individual susceptibility variants (17).

Some machine learning-based modeling approaches can aggregate small effects of multiple single nucleotide polymorphisms (SNPs) to achieve higher predictive power. Great efforts have been made to build prediction models for various human diseases, such as coronary artery disease, type 2 diabetes, and breast cancer, using a set of phenotype-associated SNPs and have achieved inspiring performance (19–22). Support vector machine and random forest (RF) are two popular machine learning algorithms that have been applied to genomic data (23, 24). Considering that machine learning models have not been used to predict smoking, we sought to explore their applicability in the development of predictive models for this behavior.

We first selected candidate SNP subsets from genome-wide imputed SNPs based on both LASSO and logistic regression. Then we used the SVM and RF algorithms to determine the SNP subset that has the highest classification performance in distinguishing smokers from nonsmokers. By comparing two machine learning algorithms and SNP subset selection methods, we aimed to build a reliable predictive model for tobacco smoking.

## Materials and Methods

### Subjects

A total of 3,371 participants of African origin were selected from the Mid-South Tobacco Case-Control (MSTCC) study population (25). All participants were recruited from the Jackson area of Mississippi during 2005–2011, and they were biologically unrelated to each other. A more detailed description of the recruitment criteria used for the MSTCC sample have been published by our group (25, 26). The recruitment of subjects and all materials used for the recruitment were approved by the Institutional Review Boards of the University of Virginia and University of Mississippi Medical Center, and informed written consent was obtained from all participants.

In the current study, 2,934 subjects (1,431 smokers and 1,503 non-smokers) recruited during 2007–2011 were randomly divided into training and test datasets, where the training dataset consisted of 70% of the subjects (N = 2,054) and the remaining 880 were the test dataset. In addition, 437 subjects (213 smokers and 224 non-smokers) recruited during 2007–2008 were used as an independent test dataset. Detailed characteristics of the datasets are presented in **Table 1**. Based on the definition of smoking commonly used in the field, all smokers had smoked at least 100 cigarettes in their lifetimes, whereas nonsmokers were required to have smoked 1–99 cigarettes but had no tobacco use in the past year (27–29).

**Table 1:** Characteristics of datasets used for machine learning.

Characteristic	Training and test samples (N = 2,934)	Independent test sample (N = 437)
Mean age (years)	42.8 ± 13.5	39.8 ± 13.3
Females (%)	1,590 (54.2)	218 (49.9)
No. smokers (%)	1,431 (48.8)	213 (48.7)

### Genotyping, Imputation, and Quality Control

For all subjects, genomic DNA was extracted from ethylenediaminetetraacetic acid (EDTA)-treated peripheral venous blood cells using the Qiagen DNA purification kit. All DNA samples were treated with RNase A to remove any contaminating RNA, and DNA quality and the concentration of each sample were determined by the A260/A280 absorbance ratio. Genotyping was conducted with the Illumina Infinium Human Exome BeadChip (Illumina Inc., San Diego, CA, US) according to the manufacturer's instructions. This chip was intended to detect association of rare variants with a larger effect size, which was developed from functional exonic variants (>90%) and disease-associated tag markers found at least three times in more than two datasets from the whole-exome sequencing of more than 12,000 individuals (www.illumina.com).

After genotyping of 242,901 SNPs from each DNA sample, we conducted a genome-wide imputation using IMPUTE2 (30) in 5-Mb chunks after pre-phasing with SHAPEIT2 (31), which yielded a total of 21,329,694 imputed SNPs. The 1000 Genomes Project (phase 3) was used as the reference panel for haplotypes (32). Following imputation, we employed a series of procedures in our SNP data quality control, which included individual-level missingness, SNP-level missingness, Hardy-Weinberg equilibrium (HWE), minor allele frequency, and population stratification (33–35). Briefly, the following quality control criteria were used to screen all SNPs: 1) a cutoff of 0.6 for the “info” metric to filter out poorly imputed variation; 2) exclusion of any SNP with a minor allele frequency (MAF) of <5% and a call rate of <95%; and 3) removal of any SNP that was not in HWE at a *P* value of <1.0 × 10^−5^. Finally, a total of 282,279 common SNPs with a MAF of >0.05 was included. Because of the requirement of the machine learning algorithms, any missing genotype of a sample was filled in with BEAGLE (v. 4.1) program (33).

We also implemented strict and rigorous quality control of each sample in both selection and population substructure assessments. The samples with incomplete phenotypic information were removed. Meanwhile, to evaluate the population structure and identify potential outliers, we performed principal components analysis (PCA) using EIGENSTRAT (36).

### Feature Selection and Risk Prediction

To be consistent with reports by others (21, 37), a straightforward approach for the numerical encoding of a genotype was assigned to 0, 1, or 2 based on the number of minor alleles. With the intention of detecting the most informative variants, we applied logistic regression with a *P* value of 0.01 or 0.05 and LASSO regression with a λ value of 10^−3^, 10^−5^, or 10^−7^ to filter out variants with less important genetic effect. After filtering, each sample was randomly assigned to either the training or test dataset, which then was used for final evaluation of the predictive power of the machine learning models.

**Figure 1:**
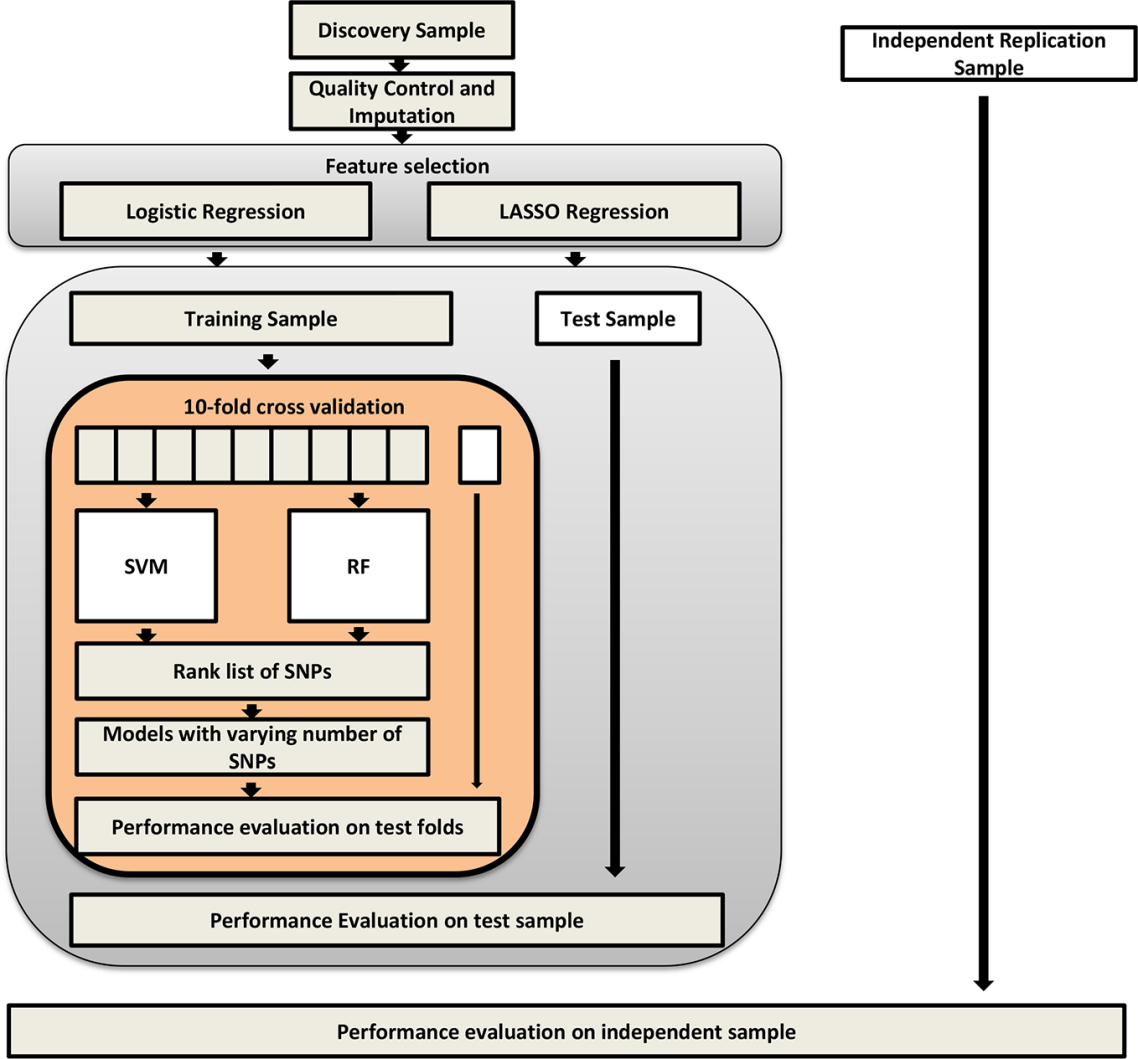
Flowchart of machine learning process used in the study.

Initially, two machine learning methods with 10-fold cross-validation were implemented to develop predictive models: support vector machine and random forest (RF) (**Figure 1**). A ranked list of all SNPs was generated at each iteration of cross-validation with SVM-recursive feature elimination (RFE) (38) or RF-RFE (39). The RFE was used to build the model with the feature selected according to the size of the eigenvalue for each SNP. Such a process was repeated over the rest of the features until all the features had been traversed. Then a list of SNPs was obtained according to the ranked mean from 10-fold cross-validations. In accordance with the rank list, the machine learning models with different numbers of top SNPs were constructed by SVM or RF. Furthermore, the test set was entered into the machine learning models to assess their performances. Finally, the independent dataset was employed to estimate the generation ability of the models using receiver operating characteristic (ROC) curves (40).

### Logistic Regression

For the remaining 282,279 SNPs, we carried out association analysis of each SNP with smoking status using a logistic regression model implemented in PLINK (v. 1.07) (41). An additive genetic model was used for association analysis for each SNP in the sample, adjusted for sex and age.

### Least Absolute Shrinkage and Selection Operator Regression

Further, we carried out association analysis of each SNP with smoking status using the least absolute shrinkage and selection operator (LASSO) (42), which minimizes the squared error with constraints on the L1 norm of β:

$$\underset{\beta }{\min}{\left(y-X\text{β}\right)}^{T}\left(y-X\text{β}\right)+\text{λ}\sum_{j=1}^{p}\left|{\text{β}}_{j}\right|,$$

where λ >0 is the tuning parameter controlling the amount of penalty (43). It shrinks the regression coefficients of some redundant features to 0 and intends to retain those features with important genetic roles in the model (42). With the large number of features we commonly face in a genomic study, we aimed to select a subset of features that exhibits a large genetic effect on the phenotype of interest (42). In the current study, we used LASSO regression with a λ value of 10^−3^, 10^−5^, or 10^−7^ to filter out variants with less important genetic effects and to retain those promising features that have non-zero coefficients using R package **glmnet** (44).

### Support Vector Machine

Support vector machine is a data mining method for regression, classification, and other pattern recognition tasks that has been used for handwritten digital recognition (45), object recognition (46), speaker identification (47), face detection (48), and bioinformatics (49). Intuitively, the method intends to find a hyperplane to separate cases from two different classes with the largest margin. The optimal separating hyperplane can be expressed formally as to minimize the functional (50):

$$\frac{1}{2}{\left\|W\right\|}^{2}+C\sum_{i=1}^{l}\xi_{i}$$

subject to constraints:

$$\left(w^{T}x_{i}+b\right)\geq 1-\xi_{i},\;i=1,\ldots ,l,$$

$${\text{ξ}}_{i}\geq 0,\;i=1,\;\ldots ,\;l,$$

Where **w** is the weights of the hyperplane, *C* is a constant, *ξ* is a non-negative slack variable, ***x*** and *y_i_* are the features and label of the cases, and *b* is the bias. This optimization process is based on large margin separation and kernel functions. The linear kernel was used to build the model:

$$\text{K}\left(x_{1},x_{2}\right)=x_{1}^{T}x_{2}$$

The R package used was **e1071** (https://CRAN.R-project.org/package=e1071). We used linear kernels by treating smokers as positive samples, nonsmokers as negative samples, and SNP genotypes as categorical features.

### Random Forest

Random forest (51) is an ensemble of decision tree features generated by bagging predictors (52). In addition to constructing each tree using a different bootstrap sample of the data, RF can determine how the classification or regression trees are constructed (53). In standard trees, each node is split using the best split among all variables (53). In an RF, each node is split using the best among a subset of predictors randomly chosen at that node (53). Each tree is a classifier and casts a unit vote for the most popular class at the input. A random selection of a subset of features governs the growth of each tree in the ensemble. For feature selection, RF models generate Mean Decrease Gini as the importance score of each feature. Any feature with a higher importance score makes a larger contribution to the model for the prediction. The “Gini importance” describes the improvement of the individual tree in the “Gini gain” splitting criterion produced by each variable (54). The **randomForest** package developed by Breiman and Cutler (https://www.stat.berkeley.edu/~breiman/RandomForests/) was used in this study.

### K-Fold Cross-Validation

In k-fold cross-validation (sometimes called rotation estimation), the dataset D is randomly split into k mutually exclusive subsets *D_1_*;*D_2_*;…;*D_k_* of approximately equal size (55). The model is trained and tested k times, and for each time *tϵ*{1,2,∙∙∙,*k*}, it is trained on all subsets except *D_1_*, and then it is tested on *D_1_*. The cross-validation estimate of accuracy is the overall number of correct classifications divided by the number of instances in the dataset (55). Specifically, 10-fold cross-validation was used in this study by randomly dividing 70% of the whole sample into 10 equal-size subsamples. Of the 10 subsamples, a single 1 was retained as the validation data for testing the model, and the remaining 9 were used as the training dataset. The cross-validation process was repeated 10 times, with each subsample being used exactly once as the validation data.

### Prediction Performance Assessment

A ROC curve was used to analyze the prediction model. Such a curve captures the trade-off between sensitivity and specificity at thresholds over a continuous range (40). The full area under the curve (AUC) measures the performance of the model. A perfect test has an AUC of 1.0, whereas random chance gives an AUC of 0.5 (40). A higher score (closer to 1) represents better discriminatory power.

## Results

We first evaluated the performance of SVM and RF with different feature (SNP) selection methods. After feature selection, the AUC of each prediction algorithm was measured by increasing the number of features from 10 to 2,000 under the consideration of clinical application for prediction. The evaluation of predictability for each machine learning model was based on the results from 10-fold cross-validation. Each SNP dataset was calculated 10 times, and the average value of AUC was used as the result of model training. Further, we exploited an independent dataset for further validating the performance of each machine learning model.

### Feature Selection

After genotyping, imputation, and quality control, 282,279 SNPs were retained as a candidate SNP set for machine learning. Both logistic and LASSO regression were used for SNP selection. We also selected a candidate SNP dataset by combining LASSO and logistic regression. The number of SNPs obtained from the feature selection with different methods and parameters is shown in **Table 2**.

**Table 2:** Number of single nucleotide polymorphisms (SNPs) selected from logistic regression, least absolute shrinkage and selection operator (LASSO) regression, and the combined logistic regression and LASSO regression under different significance thresholds.

Method	Significance thresholds	Selected SNP dataset
Logistic	*P <* 0.05	18,078
	*P <* 0.01	3,808
LASSO	λ = 10^−3^	3,518
	λ = 10^−5^	9,034
	λ = 10^−7^	46,321
Logistic + LASSO	*P <* 0.01 and λ = 10^−3^	1,149

### Performance of Support Vector Machine Models

**Figure 2:**
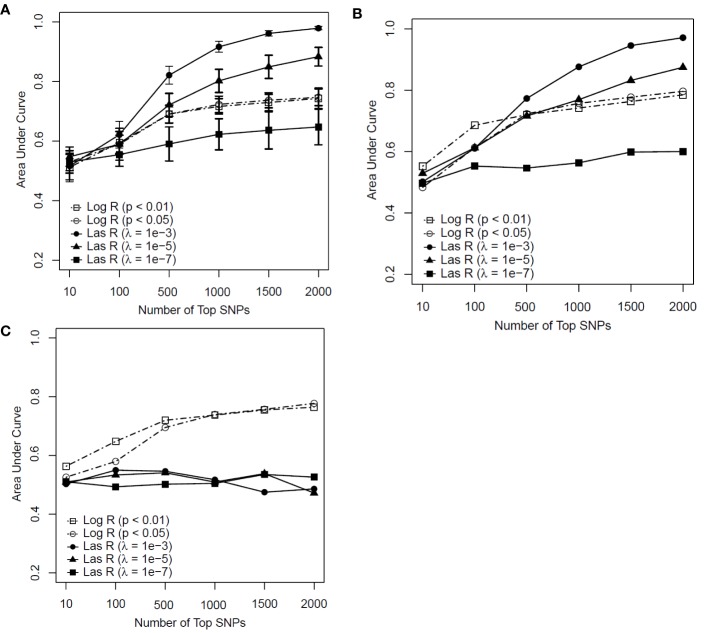
Predictive performance of support vector machine (SVM) models based on two feature selection methods with different parameters [logistic regression (Log R) and least absolute shrinkage and selection operator (LASSO) regression (Las R)]. **(A)** Evaluation of performance on training sample with 10-fold cross-validation; **(B)** evaluation of performance on test sample; and **(C)** evaluation of performance on independent test sample.

**Figure 2** shows the predictive performance of the SVM model with logistic and LASSO regression at different thresholds for feature selection by changing the number of SNPs included in the development of a predictive model for smoking. **Figure 2A** is the performance of the model determined by measuring the AUC in the training sample with the mean and standard deviation (SD) of the AUCs being calculated from a 10-fold cross-validation.

In comparing LASSO regression with logistic regression for feature selection, the performance of LASSO regression with a λ value of 10^−3^ and 10^−5^ had an advantage when more than 500 SNPs were included in the model for both the training and test samples (**Figures 2A, B**). In contrast, for the independent test sample (**Figure 2C**), the predictability of the SVM model performed less well with LASSO regression than with logistic regression for all significant thresholds and numbers of SNPs included in each model. Based on these results, we concluded that the SVM model with LASSO regression had a weak generalization ability, whereas the logistic regression worked well on both the test and independent datasets as a better feature selection method.

Regarding the SVM model with logistic regression, although an increasing trend of performance was achieved with the number of top SNPs included, there appeared to be a decreasing tendency in their performance when more than 500 SNPs were included (see **Figures 2A–C**). Taken together, the SVM model with logistic regression at a *P* value of <0.01 achieved AUCs of 0.691, 0.721, and 0.720 for the training, test, and independent test samples, respectively, on the top 500 SNPs selected for the prediction model.

### Performance of Random Forest Models

**Figure 3:**
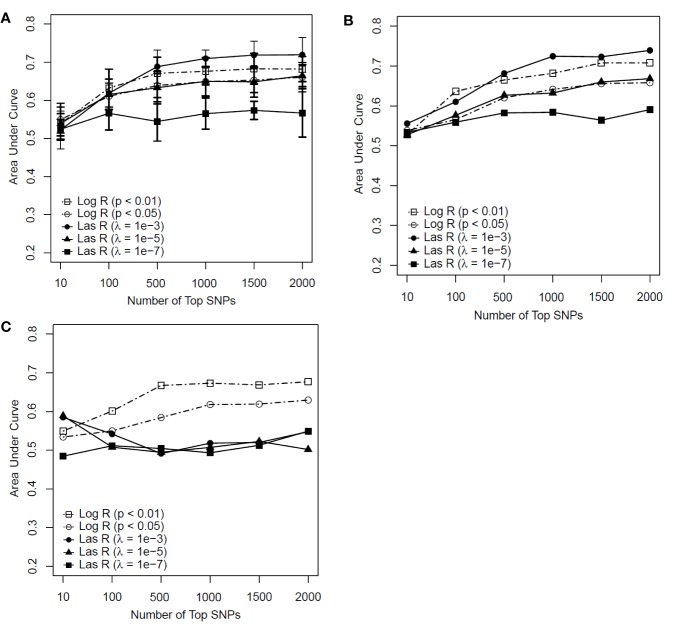
Predictive performance of random forest (RF) models based on feature selection methods with different parameters [logistic regression (Log R) and least absolute shrinkage and selection operator (LASSO) regression (Las R)]. **(A)** Evaluation of performance on training sample with 10-fold cross-validation; **(B)** evaluation of performance on test sample; and **(C)** evaluation of performance on independent test sample.

As shown in **Figures 3A, B**, the RF models with λ = 10^−3^ had an advantage over the models with other parameters when more than 500 SNPs were included for both the training and test datasets. However, for the independent test dataset, the RF model with λ = 10^−3^ had weaker predictability than those models based on logistic regression (**Figure 3C**). This indicated that the RF model with LASSO regression as a feature selection method faced the same generalization problem as the SVM model did. The RF model using logistic regression with a *P* value of <0.01 outperformed the other models (see **Figures 3A–C**). With the top 500 SNPs selected by logistic regression for inclusion, the AUC score of the model achieved 0.671, 0.665, and 0.667 for the training, test, and independent test, respectively.

### Comparison of Support Vector Machine With Random Forest Models

Based on the feature selection methods with both logistic and LASSO regression, we developed several machine learning models among five significant thresholds for feature selection. The logistic regression with a significance threshold of *P <*0.01 performed well in both the SVM and RF models, which represents the best parameter for smoking status prediction in this study. The SVM models outperformed RF models across various numbers of SNPs from 10 to 2,000 for the prediction of smoking status on both the test and independent datasets (**Table 3**; **Supplementary Tables 4** and **5**).

**Table 3:** Area under the curve (AUC) value of machine learning models utilizing logistic regression (*P <*0.01) as feature selection method.

No. of SNPs	SVM		Random forest	
	Test sample	Independent test sample	Test sample	Independent test sample
10	0.552	0.563	0.528	0.549
100	0.686	0.648	0.636	0.601
500	0.721	0.720	0.665	0.667
1,000	0.742	0.738	0.681	0.673
1,500	0.764	0.755	0.708	0.669
2,000	0.785	0.764	0.708	0.677

### Performance of Support Vector Machine Models With Combined Feature Selection Methods

**Figure 4:**
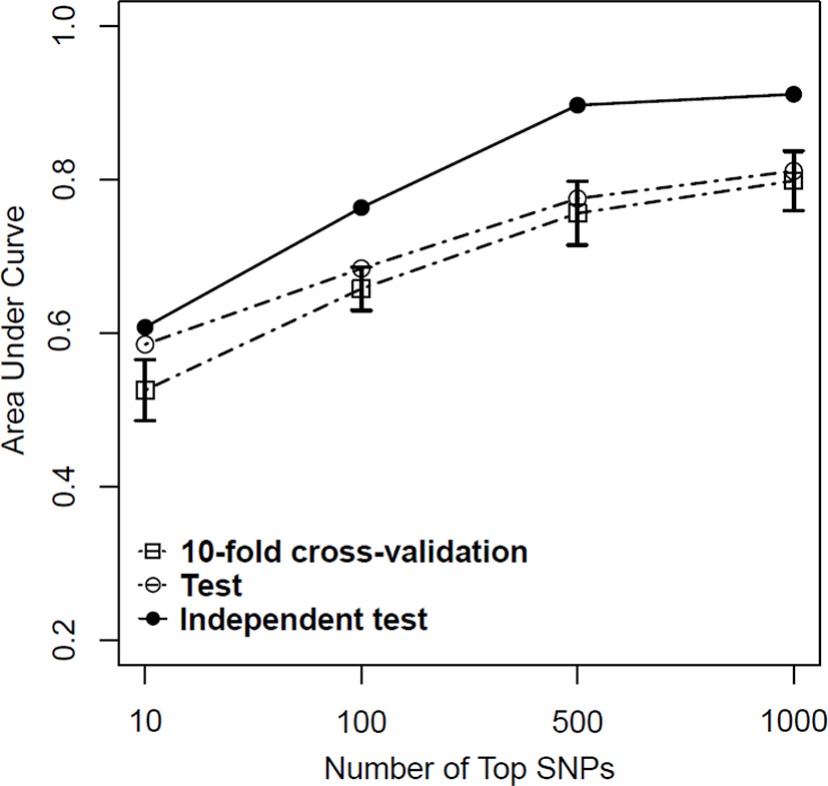
Predictive performance of support vector machine (SVM) models based on a combination of logistic regression (*P <* 0.01) and least absolute shrinkage and selection operator (LASSO) regression (λ = 10^−3^) in training, test, and independent test samples.

Based on above-mentioned results, we finally developed an SVM model using a combined feature selection approach of logistic and LASSO regression. During the feature selection process, we first used logistic regression to select those SNPs with a *P* value of <0.01 and then used LASSO regression with a λ value of <10^−3^ to complete the second stage. After completing all machine learning processes, we found that the SVM model with the combined feature selection approach of both logistic regression and LASSO regression appeared to be better than the models using only one method for both the test and independent test samples regardless of the number of SNPs (from 10 to 1,000) included in each model (**Table 4**). More importantly, we found that the AUC values did not improve significantly when the number of SNPs included in each model reached 500 (**Figure 4**).

**Table 4:** Comparison of area under the curve (AUC) values under support vector machine (SVM) model with feature selection methods of logistic regression, least absolute shrinkage and selection operator (LASSO) regression, and combined logistic and LASSO regression.

No. of SNPs	Logistic regression (*P <*0.01)		LASSO regression (λ = 10^−3^)		Logistic (*P <*0.01) + LASSO regression (λ = 10^−3^)	
	Test sample	Independent test sample	Test sample	Independent test sample	Test sample	Independent test sample
10	0.552	0.563	0.502	0.503	0.586	0.608
100	0.686	0.648	0.611	0.550	0.684	0.764
500	0.721	0.720	0.773	0.546	0.776	0.897
1,000	0.742	0.738	0.877	0.517	0.812	0.911

Given the results obtained from this series of parameter selections and machine learning methods, we concluded that the SVM model with the combined logistic regression (*P* < 0.01) and LASSO regression (λ = 10^−3^) as the feature selection method represented the best approach of developing our prediction model for the datasets used in this study. Under such condition, we achieved AUCs of 0.756, 0.776, and 0.897 for the training, test, and independent test samples, respectively.

## Discussion

Along with technological advances, experimental approaches for genetic studies on complex diseases/traits have evolved rapidly from genome-wide linkage study to candidate gene association study and from GWAS to targeted sequencing (17). Even though previous studies on tobacco smoking have revealed numerous genetic factors, the estimated heritability explained by these identified factors has been relatively small (14–18). Recent studies have indicated that machine learning approaches employed in modeling performed well in the prediction of various human diseases, such as cancer, heart disease, and Alzheimer's disease (56–58). In this study, we employed both SVM and RF approaches to develop predictive models for smoking status with both logistic and LASSO regression used for feature selection.

Although LASSO regression had an advantage in the results of training and test samples, it performed less well on the independent dataset, indicating poor generalizability. On the other hand, the machine learning models with logistic regression performed well on the independent test sample. Further, as the number of top SNPs included in the models increased from 10 to 500, the performance of the models improved under all conditions. However, such improvement attained a plateau when the top number of SNPs reached about 500. Taking all these findings together, we found that both machine learning approaches performed well on the datasets used in this study, but the SVM method appears to be superior.

Another key issue in this type of research is how many SNPs should be included in the prediction model. Although we generally believe more SNPs are better, larger numbers increase the cost of genotyping and data analysis. How to reduce the cost associated with genotyping and shorten the time required to analyze genotyped genetic data have been major concerns from the clinical point of view. This issue has become more important in today's genetic research on complex human traits, as we can easily get hundreds of thousands of SNPs genotyped for a subject of interest. To reduce the number of SNPs included in each model, both logistic and LASSO regression were used to filter out those variants with less important genetic effects on the phenotype of interest. Under these considerations, we compared the performances of all models with different numbers of top SNPs included in each model and found that a total of the top 500 SNPs included in the model appeared to be a better choice for all cases examined in this study.

Given the better performance of the SVM relative to the RF method, we developed an SVM model with the 500 top SNPs selected using a combined feature selection method of logistic regression (*P <*0.01) and LASSO regression (λ = 10^−3^). Initially, we did not expect that the predictive performance would improve significantly given that the performance of the SVM model with logistic regression had already achieved impressive results. To our surprise, we found a significant improvement in predictive performance, with an AUC of 0.897 achieved for the independent dataset.

Considering that there are so many SNPs included in each model (see **Supplementary Table 1** for a list of the top 500 SNPs included in the final model and **Supplementary Figure 1** for a comparison of the top 500 SNPs selected by logistic regression at a *P* value of <0.01 and LASSO regression with λ = 10^−3^), it is impossible as well as unreasonable to discuss them one by one. In fact, this activity probably is unnecessary, as the selection of SNPs for incorporation into our prediction model was based on the association of SNPs with smoking without considering the biological functions of the genes where these SNPs are located. Nevertheless, the first SNP rs1449123, located in the 2 KB sequence upstream of olfactory receptor family 2 subfamily D member 2 (*OR2D2*), deserves to be discussed as an example. *OR2D2* is a member of a family that produces a unique component of the signal transduction pathway involved in odorant discrimination (59). By examining the full list of SNPs included in the models, we found that 15 of the top 500 SNPs are related to olfactory receptor genes. A gene cluster for olfactory receptors is close to the MHC region on chromosome 6 (60). Füst et al. showed a potential role of the MHC-linked olfactory receptor genes in the initiation of smoking (60). Our group also reported that taster status plays a role in governing the development of nicotine dependence (ND) and may represent a way to identify individuals at risk for ND, particularly in AA smokers (61).

We also did gene ontology (GO) enrichment and pathway analyses (**Supplementary Tables 2** and **3**) based on the genes in which those top 500 SNPs are located and found many genes are related to the immune system. For example, the products of seven genes are involved in antigen presentation: folding, assembly, and peptide loading of class I MHC. The regulatory effect of smoking on immune-related pathways has been reported several times by many groups, including ours (62–64).

Technological advances can always drive the development of new approaches. Applying machine learning methods to genomic data analysis has become a promising way to dig out the complex relations between these data and the phenotype(s) of interest. Given the high AUC score of our prediction model for smoking status classification, the top 500 SNPs included in the model are assumed to contribute greatly to smoking behavior. However, the explanation for the complex relations between these top SNPs and smoking is far from complete. The main question we would like to address is why does this group of SNPs yield such a high performance for the prediction of smoking status? Our future work will try to identify the inner complex relations between these SNPs and smoking status.

There are several limitations of this study. First, compared with those reported GWAS where hundreds of thousands of samples commonly were included, the sample size used in this study was small. However, this was not a GWAS, and the objective differed from that of a GWAS. The primary goal of this study was to determine whether a machine learning approach could be used to develop a prediction model for clinical testing based on the genotyped biomarkers of a participant of interest. Under such circumstances, we believe that the sample is large enough to accomplish our goal, although we do agree that a large sample is always better. The second issue is related to the number of SNPs selected for inclusion in the model. Although it is generally believed that more SNPs would be better, it would increase genotyping cost and the computing power needed to analyze the data. Given these concerns, along with the objectives of this work, we selected a number of SNPs from 10 to 2,000 for our model constructions. Third, only the SVM and RF methods were examined in this study. It would be interesting to test other models such as elastic net for this type of research.

In sum, this study compared different methods (SVM and RF for model development; logistic regression and LASSO regression for feature selection) to build a predictive model for smoking behavior. By applying the SVM approach with a combination of logistic regression (*P <*0.01) and LASSO regression (λ = 10^−3^), we developed a statistically sound predictive model for smoking behavior. It is our hope that such a model could be used for the prevention of smoking initiation. In addition, it would be interesting to determine whether such a model could be generalized to other ethnic samples in future studies.

## Data Availability Statement

The original contributions presented in the study are publicly available. This data can be found here: NCBI GEO (https://www.ncbi.nlm.nih.gov/geo/) (accession: GSE148375).

## Ethics Statement

The studies involving human participants were reviewed and approved by the Institutional Review Boards of the University of Virginia and University of Mississippi Medical Center. The patients/participants provided their written informed consent to participate in this study.

## Author Contributions

YX, LC, XZ, YY, QL, YiM, and YuM participated in data collection and analysis. BZ, YW, JM, and TP participated in data collection. YX, LC, JM, TP, and ML participated in writing and editing of the paper. ML and LL conceived of the study and provided the funding to the study. All authors approved the final manuscript.

## Conflict of Interest

The authors declare that the research was conducted in the absence of any commercial or financial relationships that could be construed as a potential conflict of interest.
