# Supplementary material for: Prediction of Smoking Behavior From Single Nucleotide Polymorphisms With Machine Learning Approaches
Source: Front Psychiatry. 2020 May 14;11:416. doi: 10.3389/fpsyt.2020.00416 (PMC7241440; doi:10.3389/fpsyt.2020.00416)
Supplement: Supplementary file 1 [file DataSheet_1.docx]

**Supplemental Table 1: Summary information of the top 500 SNPs included in the final model**

| **Rank** | **rs ID #** | **genes** | **func annot** |
| --- | --- | --- | --- |
| 1 | rs1449123 | 1.4kb 5' of OR2D2 |  |
| 2 | rs10734655 | MRVI1 | intronic |
| 3 | rs2523761 | 20kb 3' of HLA-G |  |
| 4 | rs73686915 | 5.2kb 3' of CCDC129 |  |
| 5 | rs111950327 | LONP2 | intronic |
| 6 | rs4932230 | 5.2kb 5' of C15orf42 |  |
| 7 | rs73563529 | ZNF283 | intronic |
| 8 | rs142398023 | CENPF | intronic |
| 9 | rs66592214 | KLK3 | intronic |
| 10 | rs111615792 | TAS1R3 | missense |
| 11 | rs187680130 | MYOM2 | intronic |
| 12 | rs72748402 | 6.3kb 3' of IARS |  |
| 13 | rs75745275 | 7.9kb 3' of GCM2 |  |
| 14 | rs1813064 | CEACAM20 | intronic |
| 15 | rs2297721 | LCN15 | intronic |
| 16 | rs4910849 | 14kb 5' of OR52N1 |  |
| 17 | rs8100996 | ZNF468 | intronic |
| 18 | rs147035822 | 20kb 3' of CRISP2 |  |
| 19 | rs10487731 | 4.1kb 3' of CCDC129 | intronic |
| 20 | rs1463178 | 440bp 5' of OR2D2 |  |
| 21 | rs73686910 | CCDC129 | intronic |
| 22 | rs7559031 | 526bp 3' of PECR |  |
| 23 | rs6755934 | PECR | intronic |
| 24 | rs111780458 | PRRC2B | intronic |
| 25 | rs4776474 | 115kb 5' of C15orf50 |  |
| 26 | rs140859283 | 49kb 3' of NCRNA00252 | |
| 27 | rs12024757 | 53kb 5' of FLJ31662 |  |
| 28 | rs34160795 | TTLL12 | intronic |
| 29 | rs6115038 | TMC2 | intronic |
| 30 | rs78909326 | 19kb 5' of CYP2C9 |  |
| 31 | rs12091278 | 649bp 3' of AURKAIP1 |  |
| 32 | rs17566878 | FARP1 | intronic |
| 33 | rs141529746 | ACSBG2 | intronic |
| 34 | rs116300033 | 4.3kb 3' of PDCL2 |  |
| 35 | rs145190337 | 57kb 3' of ARL6 | intronic |
| 36 | rs7567110 | HDLBP | intronic |
| 37 | rs17678521 | KANK2 | intronic |
| 38 | rs62244286 | 8.4kb 5' of GOLGA4 |  |
| 39 | rs75132924 | TRIM69 | intronic |
| 40 | rs479835 | 3.5kb 5' of NOTCH4 |  |
| 41 | rs16892121 | AMACR | intronic |
| 42 | rs694540 | 3.7kb 5' of ALG8 |  |
| 43 | rs9277908 | 7.2kb 3' of COL11A2 |  |
| 44 | rs113512621 | CENPF | intronic |
| 45 | rs111263826 | 15kb 3' of PRSS42 |  |
| 46 | rs3759416 | CLSTN3 | intronic |
| 47 | rs934154 | 1.2kb 3' of PECR |  |
| 48 | rs116731530 | FOXM1 | intronic |
| 49 | rs3115603 | HLA-H | intronic |
| 50 | rs114964419 | ITCH | intronic |
| 51 | rs12879960 | NID2 | intronic |
| 52 | rs75134841 | VWDE | intronic |
| 53 | rs111541312 | TMC2 | intronic |
| 54 | rs11136256 | MAF1 | synonymous |
| 55 | rs10838637 | OR52N1 | synonymous |
| 56 | rs35042898 | PCLO | intronic |
| 57 | rs56156140 | C15orf58 | intronic |
| 58 | rs140093359 | 40kb 3' of BAK1 |  |
| 59 | rs73226358 | WDR36 | intronic |
| 60 | rs4500446 | NUP160 | intronic |
| 61 | rs58739919 | 576bp 5' of LY9 |  |
| 62 | rs111635187 | ALG8 | intronic |
| 63 | rs73794699 | SPINK5 | 3'-UTR |
| 64 | rs17537644 | POMT1 | intronic |
| 65 | rs77611445 | CDON | intronic |
| 66 | rs3212716 | JAK3 | synonymous |
| 67 | rs707465 | PER3 | intronic |
| 68 | rs28428768 | 2.1kb 3' of HLA-B |  |
| 69 | rs111662255 | SMPD3 | 3'-UTR |
| 70 | rs6669573 | CENPF | intronic |
| 71 | rs16941669 | ALDH2 | intronic |
| 72 | rs76706917 | FRAS1 | intronic |
| 73 | rs8176684 | ABO | intronic |
| 74 | rs61735961 | DVL1 | 3'-UTR |
| 75 | rs3132461 | 1.8kb 3' of MICB |  |
| 76 | rs73469253 | 16kb 5' of LOC120824 |  |
| 77 | rs164583 | 9.5kb 5' of SH2D1B |  |
| 78 | rs12371362 | 4.8kb 5' of OR6C75 |  |
| 79 | rs2045615 | FNBP4 | intronic |
| 80 | rs617700 | 2.8kb 5' of ALG8 |  |
| 81 | rs141289174 | 230kb 5' of ADAMTS3 |  |
| 82 | rs57079582 | CENPF | intronic |
| 83 | rs75547416 | 21kb 3' of VWDE |  |
| 84 | rs72880223 | 9.7kb 5' of LOC100507096 | |
| 85 | rs8059241 | ZNF500 | intronic |
| 86 | rs12662149 | 23kb 5' of IER3 |  |
| 87 | rs78888979 | 340bp 5' of OR5AU1 |  |
| 88 | rs3115604 | HLA-H | intronic |
| 89 | rs10402981 | 2.5kb 3' of CEACAM20 |  |
| 90 | rs562317 | ALG8 | intronic |
| 91 | rs41369548 | 16kb 3' of ANAPC4 |  |
| 92 | rs55827419 | BTN3A2 | intronic |
| 93 | rs112421769 | PRRC2B | intronic |
| 94 | rs204899 | TNXB | intronic |
| 95 | rs73571513 | 16kb 3' of HGSNAT |  |
| 96 | rs375307957 |  |  |
| 97 | rs16833214 | STAT4 | intronic |
| 98 | rs11890682 | 35kb 5' of ETAA1 |  |
| 99 | rs7113246 | PTPRJ | intronic |
| 100 | rs12436072 | HHIPL1 | 3'-UTR |
| 101 | rs6793567 | ULK4 | intronic |
| 102 | rs59921683 | OBP2A | intronic |
| 103 | rs11072112 | 118kb 5' of C15orf50 |  |
| 104 | rs79764883 | TRPM6 | intronic |
| 105 | rs12037177 | RIT1 | intronic |
| 106 | rs13345032 | 153bp 3' of CEACAM20 |  |
| 107 | rs10505202 | CSMD3 | intronic |
| 108 | rs7118279 | CCDC83 | 3'-UTR |
| 109 | rs61886576 | 560kb 5' of LRRC4C | intronic |
| 110 | rs1560713 | 1kb 3' of SLC44A2 |  |
| 111 | rs17429963 | FRAS1 | intronic |
| 112 | rs75870985 | VWDE | intronic |
| 113 | rs7947329 | 9kb 5' of LOC120824 |  |
| 114 | rs3132696 | 50bp 5' of HLA-H |  |
| 115 | rs78958815 | 23kb 3' of VWDE |  |
| 116 | rs9266008 | 4.8kb 3' of HLA-B |  |
| 117 | rs4788597 | DHODH | intronic |
| 118 | rs9495296 | REPS1 | intronic |
| 119 | rs10734656 | 3.9kb 5' of MRVI1 |  |
| 120 | rs61355869 | TBC1D1 | intronic |
| 121 | rs9277627 | HLA-DPB2 |  |
| 122 | rs78389429 | LCN8 | intronic |
| 123 | rs55897904 | GPR75-ASB3 | intronic |
| 124 | rs1611719 | 26kb 5' of HLA-H |  |
| 125 | rs111781899 | PAQR5 | intronic |
| 126 | rs10494884 | CR1L | intronic |
| 127 | rs62025051 | IQCK | intronic |
| 128 | rs1072752 | 20kb 5' of CYP2C9 |  |
| 129 | rs6771233 | 5.1kb 5' of CYP8B1 |  |
| 130 | rs144093018 | PRMT7 | intronic |
| 131 | rs9352733 | 12kb 3' of LGSN |  |
| 132 | rs4711933 | 26kb 5' of RHAG |  |
| 133 | rs17201595 | TNXB | intronic |
| 134 | rs141114087 | TMC5 | intronic |
| 135 | rs10808253 | MKLN1 | intronic |
| 136 | rs72753572 | HEMGN | intronic |
| 137 | rs147891316 | LRRFIP2 | intronic |
| 138 | rs78430278 | UBE2Q2 | intronic |
| 139 | rs79765028 | CPA5 | intronic |
| 140 | rs4690000 | ADD1 | intronic |
| 141 | rs57799281 | CENPF | intronic |
| 142 | rs1054176 | 678bp 5' of HLA-H |  |
| 143 | rs1624072 | 15kb 3' of HLA-B |  |
| 144 | rs9267577 | 6.2kb 3' of C6orf48 |  |
| 145 | rs115111825 | FRAS1 | intronic |
| 146 | rs3006451 | PGLYRP4 | intronic |
| 147 | rs588779 | 28kb 3' of ETAA1 |  |
| 148 | rs1466760 | 2.7kb 5' of MRVI1 |  |
| 149 | rs725309 | FBXW12 | intronic |
| 150 | rs725309 | FBXW12 | intronic |
| 151 | rs8041266 | FSIP1 | intronic |
| 152 | rs78061063 | PRMT7 | intronic |
| 153 | rs1987098 | STXBP5L | intronic |
| 154 | rs74134240 | HMCN1 | intronic |
| 155 | rs6563105 | RBM26 | intronic |
| 156 | rs453579 | SH2D1B | intronic |
| 157 | rs193105 | CDC20B | intronic |
| 158 | rs79847943 | PRSS46 | intronic |
| 159 | rs1800479 | APOB | intronic |
| 160 | rs590981 | ALG8 | intronic |
| 161 | rs77500425 | INPP5B | intronic |
| 162 | rs73571512 | 16kb 3' of HGSNAT |  |
| 163 | rs72910798 | 193kb 3' of LOC120824 | |
| 164 | rs187007962 | PION | intronic |
| 165 | rs73098519 | DIS3L2 | intronic |
| 166 | rs4519055 | NUP160 | intronic |
| 167 | rs73563637 | 81kb 3' of POTEA |  |
| 168 | rs2735076 | HCG9 | intronic |
| 169 | rs13326820 | ZFYVE20 | intronic |
| 170 | rs75895958 | SETX | intronic |
| 171 | rs6739471 | 39kb 5' of ETAA1 |  |
| 172 | rs3014903 | 1.2kb 3' of COG3 |  |
| 173 | rs76159452 | PRMT7 | intronic |
| 174 | rs7184969 | 22kb 5' of PRM1 |  |
| 175 | rs57375968 | 6.2kb 3' of MIR3143 |  |
| 176 | rs76601254 | CLOCK | intronic |
| 177 | rs73854539 | 3.2kb 3' of RAB33B |  |
| 178 | rs58239704 | AP2M1 | intronic |
| 179 | rs7092199 | 5kb 3' of HPS6 |  |
| 180 | rs1105944 | TNFRSF10B | intronic |
| 181 | rs75163715 | DUOX2 | 3'-UTR |
| 182 | rs7545308 | RIT1 | intronic |
| 183 | rs2290240 | 125bp 3' of MATL2963 |  |
| 184 | rs12428617 | LMO7 | intronic |
| 185 | rs115180485 | 6.6kb 3' of CSPG5 |  |
| 186 | rs17133172 | 910bp 3' of CPA1 |  |
| 187 | rs6769073 | NAALADL2 | intronic |
| 188 | rs753110 | FYCO1 | intronic |
| 189 | rs28367781 | 19kb 3' of HLA-B |  |
| 190 | rs56368800 | DNAH11 | intronic |
| 191 | rs78694716 | TNNI3 | intronic |
| 192 | rs12720737 | ACE | intronic |
| 193 | rs11714838 | LRRFIP2 | intronic |
| 194 | rs432971 | FRMPD2 | intronic |
| 195 | rs6694987 | CENPF | intronic |
| 196 | rs4605143 | 314kb 3' of TMEM114 |  |
| 197 | rs7830453 | TNFRSF10B | intronic |
| 198 | rs11728117 | FRAS1 | intronic |
| 199 | rs6788964 | NAALADL2 | intronic |
| 200 | rs2523933 | 11kb 5' of HCG9 |  |
| 201 | rs28753066 | 13kb 3' of HLA-B |  |
| 202 | rs10893080 | ZNF202 | intronic |
| 203 | rs2465552 | NSUN7 | intronic |
| 204 | rs2442422 | MMP26 | intronic |
| 205 | rs10771762 | CAPRIN2 | intronic |
| 206 | rs59305069 | ZNF44 | intronic |
| 207 | rs11265479 | 11kb 5' of LY9 |  |
| 208 | rs13333207 | PKD1L3 | intronic |
| 209 | rs788221 | YME1L1 | intronic |
| 210 | rs788221 | YME1L1 | intronic |
| 211 | rs9260746 | 8.8kb 5' of HCG9 |  |
| 212 | rs58118437 | 2.5kb 5' of SNHG7 |  |
| 213 | rs17167682 | RAPGEF6 | intronic |
| 214 | rs61997210 | MUC5B | missense |
| 215 | rs116503590 | FRAS1 | intronic |
| 216 | rs35333582 | CYP4B1 | intronic |
| 217 | rs2735057 | HLA-F-AS1 | intronic |
| 218 | rs79707176 | 7.6kb 3' of IL7R |  |
| 219 | rs3132697 | 842bp 5' of HLA-H |  |
| 220 | rs55861690 | HMCN1 | intronic |
| 221 | rs76977598 | PKD1L2 | intronic |
| 222 | rs597625 | ALG8 | intronic |
| 223 | rs192197239 | 14kb 3' of FSIP2 |  |
| 224 | rs148316049 | 30kb 3' of C6orf204 |  |
| 225 | rs148648107 | LRRFIP2 | intronic |
| 226 | rs3121196 | SH2D1B | intronic |
| 227 | rs17666424 | 11kb 3' of DARC |  |
| 228 | rs204885 | TNXB | intronic |
| 229 | rs1807609 | CHEK2 | intronic |
| 230 | rs61821495 | 3.7kb 3' of OR10J1 |  |
| 231 | rs1966887 | 10kb 5' of RNF5P1 |  |
| 232 | rs12043858 | 24kb 5' of CLIC4 |  |
| 233 | rs9265987 | 5.4kb 3' of HLA-B |  |
| 234 | rs112717456 | 81kb 3' of POTEA |  |
| 235 | rs73226356 | WDR36 | intronic |
| 236 | rs112457247 | PRMT7 | intronic |
| 237 | rs9688949 | BTN3A2 | intronic |
| 238 | rs2273433 | NID2 | intronic |
| 239 | rs79150009 | KIAA1683 | intronic |
| 240 | rs75708581 | FLJ39534 | intronic |
| 241 | rs1611568 | 588bp 5' of HCG2P7 |  |
| 242 | rs12722022 | HLA-DPB1 | synonymous |
| 243 | rs183716005 | 5kb 5' of MXRA8 |  |
| 244 | rs55823587 | A2ML1 | intronic |
| 245 | rs28752885 | 19kb 3' of HLA-B |  |
| 246 | rs12097637 | INADL | intronic |
| 247 | rs73582529 | PKD1L3 | intronic |
| 248 | rs17860365 | CELA1 | intronic |
| 249 | rs9260706 | 14kb 5' of HCG9 |  |
| 250 | rs59985162 | 28kb 3' of NPNT |  |
| 251 | rs9784201 | IGSF5 | intronic |
| 252 | rs181475855 | INTU | intronic |
| 253 | rs10923207 | 295bp 3' of TRIM45 |  |
| 254 | rs1453769 | 442kb 5' of SOX11 |  |
| 255 | rs9905565 | 2.8kb 5' of PRPSAP2 |  |
| 256 | rs78263401 | TBC1D1 | intronic |
| 257 | rs3212718 | JAK3 | intronic |
| 258 | rs7207851 | 31kb 5' of MTRNR2L1 |  |
| 259 | rs2150927 | 2kb 5' of CYP17A1 |  |
| 260 | rs73572853 | WWOX | intronic |
| 261 | rs6062210 | LAMA5 | intronic |
| 262 | rs6578608 | 1.9kb 3' of OR51B5 |  |
| 263 | rs74860879 | 23kb 5' of RHAG |  |
| 264 | rs72958833 | 17kb 3' of NPNT |  |
| 265 | rs3130641 | 52kb 5' of IER3 |  |
| 266 | rs73563635 | 81kb 3' of POTEA |  |
| 267 | rs660061 | ALG8 | intronic |
| 268 | rs6750028 | FAM82A1 | intronic |
| 269 | rs57758461 | ABCA5 | intronic |
| 270 | rs13102781 | 1Mb 5' of PCDH10 |  |
| 271 | rs10501351 | 7.4kb 5' of OR8H1 |  |
| 272 | rs11713172 | CCDC13 | intronic |
| 273 | rs7257045 | ZNF829 | intronic |
| 274 | rs9259192 | 989bp 5' of HLA-H |  |
| 275 | rs57394691 | DIXDC1 | intronic |
| 276 | rs1028006 | GABRA4 | intronic |
| 277 | rs336082 | CDC20B | intronic |
| 278 | rs17274884 | 2.7kb 3' of OR10A4 |  |
| 279 | rs17870026 | 13kb 5' of GTPBP10 |  |
| 280 | rs56148013 | 17kb 5' of PRRC2B |  |
| 281 | rs17576984 | 21kb 5' of NOTCH4 |  |
| 282 | rs76725083 | PRMT7 | intronic |
| 283 | rs2252965 | 11kb 5' of HCG9 |  |
| 284 | rs3128993 | 6.4kb 3' of HCG2P7 |  |
| 285 | rs1559172 | MUC16 | missense |
| 286 | rs11905512 | 12kb 5' of ZNF831 |  |
| 287 | rs55751523 | 28kb 5' of ZSCAN23 |  |
| 288 | rs2891229 | MIR548F1 | intronic |
| 289 | rs11650519 | SDK2 | intronic |
| 290 | rs8127561 | 711bp 3' of KRTAP19-5 | |
| 291 | rs77709410 | ZNRF3 | intronic |
| 292 | rs59980558 | RPL32P3 | intronic |
| 293 | rs9824629 | HTR3C | intronic |
| 294 | rs9265986 | 5.4kb 3' of HLA-B |  |
| 295 | rs2158286 | 16kb 3' of RPP21 |  |
| 296 | rs73286482 | KCNK15 | intronic |
| 297 | rs12465689 | STAT4 | intronic |
| 298 | rs11731012 | FRAS1 | intronic |
| 299 | rs2523402 | HLA-F-AS1 | intronic |
| 300 | rs11039102 | 14kb 5' of OR52N2 |  |
| 301 | rs9379884 | 2.8kb 3' of BTN1A1 |  |
| 302 | rs8059723 | ZNF500 | intronic |
| 303 | rs28703717 | 143kb 3' of CETN3 |  |
| 304 | rs28752884 | 19kb 3' of HLA-B |  |
| 305 | rs2386458 | TOP3A | intronic |
| 306 | rs6782423 | 31kb 3' of CCR3 |  |
| 307 | rs3812875 | PCDH20 | 5'-UTR |
| 308 | rs416258 | PLEKHA7 | intronic |
| 309 | rs3095332 | 9.9kb 5' of IER3 |  |
| 310 | rs113760118 | CENPF | intronic |
| 311 | rs372806 | RAD18 | intronic |
| 312 | rs1611156 | 3.8kb 5' of HLA-G |  |
| 313 | rs2995540 | EXO1 | intronic |
| 314 | rs12809335 | IPO8 | 5'-UTR |
| 315 | rs77739592 | 2.5kb 5' of DSG1 |  |
| 316 | rs12140604 | 244kb 5' of DUSP10 |  |
| 317 | rs113293848 | 70kb 3' of POTEA |  |
| 318 | rs11698130 | PABPC1L | intronic |
| 319 | rs55653538 | HMCN1 | intronic |
| 320 | rs78117607 | 4.8kb 5' of SLC22A25 |  |
| 321 | rs3816943 | TECPR1 | intronic |
| 322 | rs79459890 | GPR98 | intronic |
| 323 | rs73063735 | RNF212 | intronic |
| 324 | rs28625134 | FSIP1 | intronic |
| 325 | rs60409583 | 17kb 5' of PRM1 |  |
| 326 | rs111368654 | PRMT7 | intronic |
| 327 | rs1233376 | 4.5kb 5' of GABBR1 |  |
| 328 | rs2734970 | 21kb 5' of HLA-H |  |
| 329 | rs3827840 | OBP2A | intronic |
| 330 | rs2523504 | ATP6V1G2-DDX39B | intronic |
| 331 | rs6983549 | 7.1kb 5' of POU5F1B |  |
| 332 | rs147912107 | INPP5B | intronic |
| 333 | rs11881349 | 4.8kb 3' of ZNF44 |  |
| 334 | rs73311253 | 128bp 5' of KRT36 |  |
| 335 | rs297597 | GPD2 | intronic |
| 336 | rs17467211 | LOC285965 | intronic |
| 337 | rs9259329 | 2.3kb 3' of HLA-H |  |
| 338 | rs879586 | 13kb 3' of ANAPC4 |  |
| 339 | rs11865403 | IQCK | intronic |
| 340 | rs11727137 | FRAS1 | intronic |
| 341 | rs62407543 | 16kb 5' of HLA-L |  |
| 342 | rs1655894 | HLA-A | intronic |
| 343 | rs2844825 | 22kb 5' of HLA-H |  |
| 344 | rs113094827 | TMEM194A | intronic |
| 345 | rs4845363 | NUP210L | intronic |
| 346 | rs4713233 | 23kb 5' of ZFP57 |  |
| 347 | rs12429534 | LMO7 | intronic |
| 348 | rs1611284 | 12kb 5' of IFITM4P |  |
| 349 | rs6931952 | 14kb 5' of HCG9 |  |
| 350 | rs9815103 | FBXW12 | 5'-UTR |
| 351 | rs56077880 | CSMD3 | intronic |
| 352 | rs13142939 | C4orf17 | intronic |
| 353 | rs116371893 | 5.5kb 5' of BTN3A2 |  |
| 354 | rs896858 | 36kb 5' of EHF |  |
| 355 | rs112934253 | 4kb 5' of MXRA8 |  |
| 356 | rs112536786 | FMNL3 | intronic |
| 357 | rs9488865 | NT5DC1 | intronic |
| 358 | rs3094645 | 1.4kb 5' of HLA-H |  |
| 359 | rs725310 | FBXW12 | intronic |
| 360 | rs11722061 | FRAS1 | intronic |
| 361 | rs3093952 | MICB | intronic |
| 362 | rs113403743 | 11kb 5' of CCR2 |  |
| 363 | rs72955025 | 10kb 5' of UPK1B |  |
| 364 | rs1632878 | 4.4kb 3' of HLA-A |  |
| 365 | rs28752883 | 19kb 3' of HLA-B |  |
| 366 | rs150317970 | 66kb 3' of POTEA |  |
| 367 | rs7856857 | GRIN3A | intronic |
| 368 | rs241442 | TAP2 | intronic |
| 369 | rs7293751 | 196kb 3' of POTEA |  |
| 370 | rs148016395 | 13kb 5' of LY9 |  |
| 371 | rs11821654 | CCDC83 | intronic |
| 372 | rs17204501 | MED12L | intronic |
| 373 | rs8131342 | 544bp 3' of KRTAP19-5 | |
| 374 | rs10050652 | 855bp 5' of WDR36 |  |
| 375 | rs62413583 | 16kb 3' of CRISP2 |  |
| 376 | rs73037407 | ANO2 | intronic |
| 377 | rs36092070 | ZNF589 | intronic |
| 378 | rs1372175 | CNGB3 | intronic |
| 379 | rs9941787 | 81bp 3' of RRP1B |  |
| 380 | rs80325263 | PRMT7 | intronic |
| 381 | rs222445 | FBXO9 | intronic |
| 382 | rs17663710 | VWC2 | intronic |
| 383 | rs113057932 | SP1 | intronic |
| 384 | rs72720116 | 6.2kb 5' of CLCA2 |  |
| 385 | rs3907218 | CCDC13 | intronic |
| 386 | rs111562988 | RPLP2 | intronic |
| 387 | rs2430647 | TEKT5 | intronic |
| 388 | rs111884709 | CENPF | intronic |
| 389 | rs56117556 | MUC5B | synonymous |
| 390 | rs114956797 | FRAS1 | synonymous |
| 391 | rs8176734 | ABO | intronic |
| 392 | rs71474932 | 1.3kb 3' of OVCH2 | intronic |
| 393 | rs9261143 | ZNRD1-AS1 | intronic |
| 394 | rs4757979 | 1.5kb 3' of OR10A4 |  |
| 395 | rs75261286 | CLOCK | intronic |
| 396 | rs57771713 | FRAS1 | intronic |
| 397 | rs10897397 | SLC22A25 | intronic |
| 398 | rs73404108 | PRR14L | intronic |
| 399 | rs77587927 | 30kb 5' of PRRC2B |  |
| 400 | rs74981701 | SLC7A6OS | intronic |
| 401 | rs1632958 | 2.5kb 5' of HLA-F |  |
| 402 | rs11574431 | 1.1kb 5' of CCRL2 |  |
| 403 | rs2442723 | 1.5kb 3' of HLA-B |  |
| 404 | rs7322901 | RBM26 | intronic |
| 405 | rs11757098 | MUT | 3'-UTR |
| 406 | rs9261425 | 240bp 5' of TRIM31 |  |
| 407 | rs10458231 | 84kb 3' of LOC100506207 | |
| 408 | rs146033453 | VMAC | intronic |
| 409 | rs17860378 | 320bp 3' of CELA1 |  |
| 410 | rs10947058 | TRIM26 | intronic |
| 411 | rs12643662 | 803kb 3' of LOC100144602 | |
| 412 | rs2698763 | FRMPD2 | intronic |
| 413 | rs6090704 | NCOA3 | intronic |
| 414 | rs77111379 | DSG1 | intronic |
| 415 | rs11231405 | SLC22A25 | intronic |
| 416 | rs3094646 | 2.5kb 5' of HLA-H |  |
| 417 | rs73184342 | 5.6kb 3' of UTP14C | intronic |
| 418 | rs140230871 | 65kb 3' of POTEA |  |
| 419 | rs8186257 | 201kb 3' of LOC646813 | |
| 420 | rs145936889 | PRRC2B | intronic |
| 421 | rs966364 | EDARADD | intronic |
| 422 | rs139659694 | 303kb 3' of OR4A5 |  |
| 423 | rs4840922 | SGK223 | intronic |
| 424 | rs146579687 | 7.3kb 5' of CCRL2 |  |
| 425 | rs66757203 | 1.3kb 3' of BTN3A3 |  |
| 426 | rs116672134 | 18kb 3' of IL1F10 |  |
| 427 | rs8079586 | RNF213 | intronic |
| 428 | rs2743930 | 6.8kb 5' of HLA-G |  |
| 429 | rs73383248 | SLC17A1 | intronic |
| 430 | rs35313666 | 9.1kb 5' of RNF5P1 |  |
| 431 | rs35816125 | LIPG | intronic |
| 432 | rs35388100 | IPO8 | intronic |
| 433 | rs77530144 | 36kb 5' of HLA-C |  |
| 434 | rs79728700 | FRAS1 | intronic |
| 435 | rs9349797 | CCDC90A | intronic |
| 436 | rs2394162 | HLA-F-AS1 | intronic |
| 437 | rs11145853 | 3.8kb 3' of LCN10 |  |
| 438 | rs10265056 | VWDE | intronic |
| 439 | rs10265056 | VWDE | intronic |
| 440 | rs34390600 | NID2 | intronic |
| 441 | rs2465570 | NSUN7 | synonymous |
| 442 | rs77330900 | ZNF45 | intronic |
| 443 | rs10013743 | ANK2 | synonymous |
| 444 | rs72923107 | 87bp 3' of OR10A2 |  |
| 445 | rs112459005 | CENPF | intronic |
| 446 | rs2571408 | 9.9kb 3' of HCG2P7 |  |
| 447 | rs9260743 | 9.3kb 5' of HCG9 |  |
| 448 | rs9259141 | 3.2kb 5' of HLA-H |  |
| 449 | rs12609128 | ZNF44 | intronic |
| 450 | rs114877991 | 37kb 5' of SPEF2 |  |
| 451 | rs12019102 | 13kb 5' of CXXC1 |  |
| 452 | rs11053853 | 697bp 3' of RBP5 |  |
| 453 | rs2735053 | HLA-F-AS1 | intronic |
| 454 | rs4890009 | RNF213 | synonymous |
| 455 | rs114444027 | MXRA8 | intronic |
| 456 | rs142883156 | MUC5B | synonymous |
| 457 | rs35247001 | 26kb 5' of EPC1 |  |
| 458 | rs76089559 | WDSUB1 | intronic |
| 459 | rs9265984 | 5.5kb 3' of HLA-B |  |
| 460 | rs2596508 | 3.2kb 3' of HLA-B |  |
| 461 | rs4340318 | 314kb 3' of TMEM114 |  |
| 462 | rs588678 | CWF19L2 | intronic |
| 463 | rs9393725 | 3kb 5' of LOC285819 |  |
| 464 | rs2634023 | TGFBR3 | intronic |
| 465 | rs114876907 | SLC25A45 | 3'-UTR |
| 466 | rs74515642 | SLC7A6OS | intronic |
| 467 | rs9981889 | PCNT | intronic |
| 468 | rs9369892 | 45kb 3' of MUT |  |
| 469 | rs843570 | PRDM5 | intronic |
| 470 | rs112044314 | WWOX | intronic |
| 471 | rs55812705 | REV3L | intronic |
| 472 | rs191524270 | 11kb 3' of HLA-B |  |
| 473 | rs6656501 | MSH4 | intronic |
| 474 | rs34437200 | 17kb 5' of PRM1 |  |
| 475 | rs1737046 | 17kb 5' of IFITM4P |  |
| 476 | rs2523972 | 5.1kb 5' of HCG9 |  |
| 477 | rs3212719 | JAK3 | intronic |
| 478 | rs2332008 | 262bp 5' of SMR3B |  |
| 479 | rs71395857 | SMPD3 | intronic |
| 480 | rs4267675 | DOCK3 | intronic |
| 481 | rs4334346 | 42kb 5' of MTRNR2L1 |  |
| 482 | rs3115612 | 3.9kb 5' of HLA-H |  |
| 483 | rs113327736 | INPP5B | intronic |
| 484 | rs4947256 | 30kb 5' of TRIM27 |  |
| 485 | rs4652205 | ASTN1 | intronic |
| 486 | rs6770787 | FLJ39534 | intronic |
| 487 | rs1840175 | 12kb 5' of OR52N2 |  |
| 488 | rs11990815 | 64kb 3' of POTEA |  |
| 489 | rs73400251 | SFI1 | intronic |
| 490 | rs7543039 | 62kb 5' of PALMD |  |
| 491 | rs28591612 | TNNI3 | intronic |
| 492 | rs7945138 | 9.3kb 3' of OR8D4 |  |
| 493 | rs78703346 | PRMT7 | intronic |
| 494 | rs12079950 | MSH4 | intronic |
| 495 | rs56389663 | ABCA5 | intronic |
| 496 | rs58514600 | 34kb 5' of BRSK2 |  |
| 497 | rs3828681 | PCDHGB8P | intronic |
| 498 | rs2252997 | SLC29A3 | synonymous |
| 499 | rs61493618 | LRRFIP2 | intronic |
| 500 | rs11039417 | NUP160 | intronic |

**Supplemental Table 2: Summary information of the GO analysis**

|  | **ID** | **Name** | **p-value** | **Bonferroni-corrected p-value** | **Genes from input** | **Genes in Annotation** |
| --- | --- | --- | --- | --- | --- | --- |
| 1 | GO:0005509 | calcium ion binding | 1.18E-08 | 1.13E-05 | [42](https://toppgene.cchmc.org/showQueryTerms.jsp?userdata_id=f345aaf3-1290-4655-affc-702083b2cfb3&feature=gof&row=0) | [708](https://toppgene.cchmc.org/showTermDetail.jsp?userdata_id=f345aaf3-1290-4655-affc-702083b2cfb3&category=GeneOntologyMolecularFunction&id=GO:0005509) |
| 2 | GO:0042605 | peptide antigen binding | 2.46E-08 | 2.36E-05 | [9](https://toppgene.cchmc.org/showQueryTerms.jsp?userdata_id=f345aaf3-1290-4655-affc-702083b2cfb3&feature=gof&row=1) | [32](https://toppgene.cchmc.org/showTermDetail.jsp?userdata_id=f345aaf3-1290-4655-affc-702083b2cfb3&category=GeneOntologyMolecularFunction&id=GO:0042605) |
| 3 | GO:0046977 | TAP binding | 1.16E-07 | 1.11E-04 | [5](https://toppgene.cchmc.org/showQueryTerms.jsp?userdata_id=f345aaf3-1290-4655-affc-702083b2cfb3&feature=gof&row=2) | [7](https://toppgene.cchmc.org/showTermDetail.jsp?userdata_id=f345aaf3-1290-4655-affc-702083b2cfb3&category=GeneOntologyMolecularFunction&id=GO:0046977) |
| 4 | GO:0003823 | antigen binding | 1.74E-05 | 1.67E-02 | [12](https://toppgene.cchmc.org/showQueryTerms.jsp?userdata_id=f345aaf3-1290-4655-affc-702083b2cfb3&feature=gof&row=3) | [120](https://toppgene.cchmc.org/showTermDetail.jsp?userdata_id=f345aaf3-1290-4655-affc-702083b2cfb3&category=GeneOntologyMolecularFunction&id=GO:0003823) |
| 5 | GO:0046979 | TAP2 binding | 4.49E-05 | 4.30E-02 | [3](https://toppgene.cchmc.org/showQueryTerms.jsp?userdata_id=f345aaf3-1290-4655-affc-702083b2cfb3&feature=gof&row=4) | [4](https://toppgene.cchmc.org/showTermDetail.jsp?userdata_id=f345aaf3-1290-4655-affc-702083b2cfb3&category=GeneOntologyMolecularFunction&id=GO:0046979) |
| 6 | GO:0046978 | TAP1 binding | 4.49E-05 | 4.30E-02 | [3](https://toppgene.cchmc.org/showQueryTerms.jsp?userdata_id=f345aaf3-1290-4655-affc-702083b2cfb3&feature=gof&row=5) | [4](https://toppgene.cchmc.org/showTermDetail.jsp?userdata_id=f345aaf3-1290-4655-affc-702083b2cfb3&category=GeneOntologyMolecularFunction&id=GO:0046978) |
| 7 | GO:0007156 | homophilic cell adhesion via plasma membrane adhesion molecules | 2.64E-13 | 1.10E-09 | [24](https://toppgene.cchmc.org/showQueryTerms.jsp?userdata_id=f345aaf3-1290-4655-affc-702083b2cfb3&feature=gop&row=0) | [160](https://toppgene.cchmc.org/showTermDetail.jsp?userdata_id=f345aaf3-1290-4655-affc-702083b2cfb3&category=GeneOntologyBiologicalProcess&id=GO:0007156) |
| 8 | GO:0098742 | cell-cell adhesion via plasma-membrane adhesion molecules | 1.19E-10 | 4.95E-07 | [25](https://toppgene.cchmc.org/showQueryTerms.jsp?userdata_id=f345aaf3-1290-4655-affc-702083b2cfb3&feature=gop&row=1) | [230](https://toppgene.cchmc.org/showTermDetail.jsp?userdata_id=f345aaf3-1290-4655-affc-702083b2cfb3&category=GeneOntologyBiologicalProcess&id=GO:0098742) |
| 9 | GO:0098609 | cell-cell adhesion | 1.37E-08 | 5.70E-05 | [52](https://toppgene.cchmc.org/showQueryTerms.jsp?userdata_id=f345aaf3-1290-4655-affc-702083b2cfb3&feature=gop&row=2) | [980](https://toppgene.cchmc.org/showTermDetail.jsp?userdata_id=f345aaf3-1290-4655-affc-702083b2cfb3&category=GeneOntologyBiologicalProcess&id=GO:0098609) |
| 10 | GO:0002480 | antigen processing and presentation of exogenous peptide antigen via MHC class I, TAP-independent | 7.15E-07 | 2.97E-03 | [5](https://toppgene.cchmc.org/showQueryTerms.jsp?userdata_id=f345aaf3-1290-4655-affc-702083b2cfb3&feature=gop&row=3) | [9](https://toppgene.cchmc.org/showTermDetail.jsp?userdata_id=f345aaf3-1290-4655-affc-702083b2cfb3&category=GeneOntologyBiologicalProcess&id=GO:0002480) |
| 11 | GO:0007155 | cell adhesion | 1.70E-06 | 7.08E-03 | [64](https://toppgene.cchmc.org/showQueryTerms.jsp?userdata_id=f345aaf3-1290-4655-affc-702083b2cfb3&feature=gop&row=4) | [1530](https://toppgene.cchmc.org/showTermDetail.jsp?userdata_id=f345aaf3-1290-4655-affc-702083b2cfb3&category=GeneOntologyBiologicalProcess&id=GO:0007155) |
| 12 | GO:0022610 | biological adhesion | 2.21E-06 | 9.20E-03 | [64](https://toppgene.cchmc.org/showQueryTerms.jsp?userdata_id=f345aaf3-1290-4655-affc-702083b2cfb3&feature=gop&row=5) | [1542](https://toppgene.cchmc.org/showTermDetail.jsp?userdata_id=f345aaf3-1290-4655-affc-702083b2cfb3&category=GeneOntologyBiologicalProcess&id=GO:0022610) |
| 13 | GO:0002698 | negative regulation of immune effector process | 2.54E-06 | 1.06E-02 | [13](https://toppgene.cchmc.org/showQueryTerms.jsp?userdata_id=f345aaf3-1290-4655-affc-702083b2cfb3&feature=gop&row=6) | [116](https://toppgene.cchmc.org/showTermDetail.jsp?userdata_id=f345aaf3-1290-4655-affc-702083b2cfb3&category=GeneOntologyBiologicalProcess&id=GO:0002698) |
| 14 | GO:0001911 | negative regulation of leukocyte mediated cytotoxicity | 5.61E-06 | 2.33E-02 | [6](https://toppgene.cchmc.org/showQueryTerms.jsp?userdata_id=f345aaf3-1290-4655-affc-702083b2cfb3&feature=gop&row=7) | [21](https://toppgene.cchmc.org/showTermDetail.jsp?userdata_id=f345aaf3-1290-4655-affc-702083b2cfb3&category=GeneOntologyBiologicalProcess&id=GO:0001911) |
| 15 | GO:0042270 | protection from natural killer cell mediated cytotoxicity | 8.95E-06 | 3.72E-02 | [4](https://toppgene.cchmc.org/showQueryTerms.jsp?userdata_id=f345aaf3-1290-4655-affc-702083b2cfb3&feature=gop&row=8) | [7](https://toppgene.cchmc.org/showTermDetail.jsp?userdata_id=f345aaf3-1290-4655-affc-702083b2cfb3&category=GeneOntologyBiologicalProcess&id=GO:0042270) |
| 16 | GO:0031342 | negative regulation of cell killing | 1.31E-05 | 5.45E-02 | [6](https://toppgene.cchmc.org/showQueryTerms.jsp?userdata_id=f345aaf3-1290-4655-affc-702083b2cfb3&feature=gop&row=9) | [24](https://toppgene.cchmc.org/showTermDetail.jsp?userdata_id=f345aaf3-1290-4655-affc-702083b2cfb3&category=GeneOntologyBiologicalProcess&id=GO:0031342) |
| 17 | GO:0002483 | antigen processing and presentation of endogenous peptide antigen | 2.17E-05 | 9.02E-02 | [5](https://toppgene.cchmc.org/showQueryTerms.jsp?userdata_id=f345aaf3-1290-4655-affc-702083b2cfb3&feature=gop&row=10) | [16](https://toppgene.cchmc.org/showTermDetail.jsp?userdata_id=f345aaf3-1290-4655-affc-702083b2cfb3&category=GeneOntologyBiologicalProcess&id=GO:0002483) |
| 18 | GO:0060333 | interferon-gamma-mediated signaling pathway | 2.39E-05 | 9.94E-02 | [10](https://toppgene.cchmc.org/showQueryTerms.jsp?userdata_id=f345aaf3-1290-4655-affc-702083b2cfb3&feature=gop&row=11) | [85](https://toppgene.cchmc.org/showTermDetail.jsp?userdata_id=f345aaf3-1290-4655-affc-702083b2cfb3&category=GeneOntologyBiologicalProcess&id=GO:0060333) |
| 19 | GO:0045953 | negative regulation of natural killer cell mediated cytotoxicity | 3.02E-05 | 1.25E-01 | [5](https://toppgene.cchmc.org/showQueryTerms.jsp?userdata_id=f345aaf3-1290-4655-affc-702083b2cfb3&feature=gop&row=12) | [17](https://toppgene.cchmc.org/showTermDetail.jsp?userdata_id=f345aaf3-1290-4655-affc-702083b2cfb3&category=GeneOntologyBiologicalProcess&id=GO:0045953) |
| 20 | GO:0002484 | antigen processing and presentation of endogenous peptide antigen via MHC class I via ER pathway | 3.11E-05 | 1.29E-01 | [4](https://toppgene.cchmc.org/showQueryTerms.jsp?userdata_id=f345aaf3-1290-4655-affc-702083b2cfb3&feature=gop&row=13) | [9](https://toppgene.cchmc.org/showTermDetail.jsp?userdata_id=f345aaf3-1290-4655-affc-702083b2cfb3&category=GeneOntologyBiologicalProcess&id=GO:0002484) |
| 21 | GO:0002704 | negative regulation of leukocyte mediated immunity | 3.33E-05 | 1.39E-01 | [8](https://toppgene.cchmc.org/showQueryTerms.jsp?userdata_id=f345aaf3-1290-4655-affc-702083b2cfb3&feature=gop&row=14) | [55](https://toppgene.cchmc.org/showTermDetail.jsp?userdata_id=f345aaf3-1290-4655-affc-702083b2cfb3&category=GeneOntologyBiologicalProcess&id=GO:0002704) |
| 22 | GO:0002716 | negative regulation of natural killer cell mediated immunity | 4.10E-05 | 1.70E-01 | [5](https://toppgene.cchmc.org/showQueryTerms.jsp?userdata_id=f345aaf3-1290-4655-affc-702083b2cfb3&feature=gop&row=15) | [18](https://toppgene.cchmc.org/showTermDetail.jsp?userdata_id=f345aaf3-1290-4655-affc-702083b2cfb3&category=GeneOntologyBiologicalProcess&id=GO:0002716) |
| 23 | GO:0019883 | antigen processing and presentation of endogenous antigen | 5.46E-05 | 2.27E-01 | [5](https://toppgene.cchmc.org/showQueryTerms.jsp?userdata_id=f345aaf3-1290-4655-affc-702083b2cfb3&feature=gop&row=16) | [19](https://toppgene.cchmc.org/showTermDetail.jsp?userdata_id=f345aaf3-1290-4655-affc-702083b2cfb3&category=GeneOntologyBiologicalProcess&id=GO:0019883) |
| 24 | GO:0002474 | antigen processing and presentation of peptide antigen via MHC class I | 5.76E-05 | 2.39E-01 | [10](https://toppgene.cchmc.org/showQueryTerms.jsp?userdata_id=f345aaf3-1290-4655-affc-702083b2cfb3&feature=gop&row=17) | [94](https://toppgene.cchmc.org/showTermDetail.jsp?userdata_id=f345aaf3-1290-4655-affc-702083b2cfb3&category=GeneOntologyBiologicalProcess&id=GO:0002474) |
| 25 | GO:0019221 | cytokine-mediated signaling pathway | 8.00E-05 | 3.32E-01 | [28](https://toppgene.cchmc.org/showQueryTerms.jsp?userdata_id=f345aaf3-1290-4655-affc-702083b2cfb3&feature=gop&row=18) | [553](https://toppgene.cchmc.org/showTermDetail.jsp?userdata_id=f345aaf3-1290-4655-affc-702083b2cfb3&category=GeneOntologyBiologicalProcess&id=GO:0019221) |
| 26 | GO:0050777 | negative regulation of immune response | 9.16E-05 | 3.81E-01 | [12](https://toppgene.cchmc.org/showQueryTerms.jsp?userdata_id=f345aaf3-1290-4655-affc-702083b2cfb3&feature=gop&row=19) | [140](https://toppgene.cchmc.org/showTermDetail.jsp?userdata_id=f345aaf3-1290-4655-affc-702083b2cfb3&category=GeneOntologyBiologicalProcess&id=GO:0050777) |
| 27 | GO:0048002 | antigen processing and presentation of peptide antigen | 9.50E-05 | 3.95E-01 | [14](https://toppgene.cchmc.org/showQueryTerms.jsp?userdata_id=f345aaf3-1290-4655-affc-702083b2cfb3&feature=gop&row=20) | [185](https://toppgene.cchmc.org/showTermDetail.jsp?userdata_id=f345aaf3-1290-4655-affc-702083b2cfb3&category=GeneOntologyBiologicalProcess&id=GO:0048002) |
| 28 | GO:0002478 | antigen processing and presentation of exogenous peptide antigen | 1.19E-04 | 4.94E-01 | [13](https://toppgene.cchmc.org/showQueryTerms.jsp?userdata_id=f345aaf3-1290-4655-affc-702083b2cfb3&feature=gop&row=21) | [166](https://toppgene.cchmc.org/showTermDetail.jsp?userdata_id=f345aaf3-1290-4655-affc-702083b2cfb3&category=GeneOntologyBiologicalProcess&id=GO:0002478) |
| 29 | GO:0019884 | antigen processing and presentation of exogenous antigen | 1.80E-04 | 7.47E-01 | [13](https://toppgene.cchmc.org/showQueryTerms.jsp?userdata_id=f345aaf3-1290-4655-affc-702083b2cfb3&feature=gop&row=22) | [173](https://toppgene.cchmc.org/showTermDetail.jsp?userdata_id=f345aaf3-1290-4655-affc-702083b2cfb3&category=GeneOntologyBiologicalProcess&id=GO:0019884) |
| 30 | GO:0019885 | antigen processing and presentation of endogenous peptide antigen via MHC class I | 2.25E-04 | 9.36E-01 | [4](https://toppgene.cchmc.org/showQueryTerms.jsp?userdata_id=f345aaf3-1290-4655-affc-702083b2cfb3&feature=gop&row=23) | [14](https://toppgene.cchmc.org/showTermDetail.jsp?userdata_id=f345aaf3-1290-4655-affc-702083b2cfb3&category=GeneOntologyBiologicalProcess&id=GO:0019885) |
| 31 | GO:0001913 | T cell mediated cytotoxicity | 2.38E-04 | 9.89E-01 | [6](https://toppgene.cchmc.org/showQueryTerms.jsp?userdata_id=f345aaf3-1290-4655-affc-702083b2cfb3&feature=gop&row=24) | [39](https://toppgene.cchmc.org/showTermDetail.jsp?userdata_id=f345aaf3-1290-4655-affc-702083b2cfb3&category=GeneOntologyBiologicalProcess&id=GO:0001913) |
| 32 | GO:0019882 | antigen processing and presentation | 3.08E-04 | 1.00E+00 | [15](https://toppgene.cchmc.org/showQueryTerms.jsp?userdata_id=f345aaf3-1290-4655-affc-702083b2cfb3&feature=gop&row=25) | [232](https://toppgene.cchmc.org/showTermDetail.jsp?userdata_id=f345aaf3-1290-4655-affc-702083b2cfb3&category=GeneOntologyBiologicalProcess&id=GO:0019882) |
| 33 | GO:0042611 | MHC protein complex | 1.05E-07 | 5.55E-05 | [8](https://toppgene.cchmc.org/showQueryTerms.jsp?userdata_id=f345aaf3-1290-4655-affc-702083b2cfb3&feature=goc&row=0) | [27](https://toppgene.cchmc.org/showTermDetail.jsp?userdata_id=f345aaf3-1290-4655-affc-702083b2cfb3&category=GeneOntologyCellularComponent&id=GO:0042611) |
| 34 | GO:0042612 | MHC class I protein complex | 2.50E-06 | 1.32E-03 | [5](https://toppgene.cchmc.org/showQueryTerms.jsp?userdata_id=f345aaf3-1290-4655-affc-702083b2cfb3&feature=goc&row=1) | [11](https://toppgene.cchmc.org/showTermDetail.jsp?userdata_id=f345aaf3-1290-4655-affc-702083b2cfb3&category=GeneOntologyCellularComponent&id=GO:0042612) |
| 35 | GO:0071556 | integral component of lumenal side of endoplasmic reticulum membrane | 3.11E-06 | 1.65E-03 | [7](https://toppgene.cchmc.org/showQueryTerms.jsp?userdata_id=f345aaf3-1290-4655-affc-702083b2cfb3&feature=goc&row=2) | [29](https://toppgene.cchmc.org/showTermDetail.jsp?userdata_id=f345aaf3-1290-4655-affc-702083b2cfb3&category=GeneOntologyCellularComponent&id=GO:0071556) |
| 36 | GO:0098553 | lumenal side of endoplasmic reticulum membrane | 3.11E-06 | 1.65E-03 | [7](https://toppgene.cchmc.org/showQueryTerms.jsp?userdata_id=f345aaf3-1290-4655-affc-702083b2cfb3&feature=goc&row=3) | [29](https://toppgene.cchmc.org/showTermDetail.jsp?userdata_id=f345aaf3-1290-4655-affc-702083b2cfb3&category=GeneOntologyCellularComponent&id=GO:0098553) |
| 37 | GO:0098576 | lumenal side of membrane | 7.85E-06 | 4.16E-03 | [7](https://toppgene.cchmc.org/showQueryTerms.jsp?userdata_id=f345aaf3-1290-4655-affc-702083b2cfb3&feature=goc&row=4) | [33](https://toppgene.cchmc.org/showTermDetail.jsp?userdata_id=f345aaf3-1290-4655-affc-702083b2cfb3&category=GeneOntologyCellularComponent&id=GO:0098576) |
| 38 | GO:0009986 | cell surface | 2.31E-04 | 1.23E-01 | [37](https://toppgene.cchmc.org/showQueryTerms.jsp?userdata_id=f345aaf3-1290-4655-affc-702083b2cfb3&feature=goc&row=5) | [873](https://toppgene.cchmc.org/showTermDetail.jsp?userdata_id=f345aaf3-1290-4655-affc-702083b2cfb3&category=GeneOntologyCellularComponent&id=GO:0009986) |
| 39 | GO:0012507 | ER to Golgi transport vesicle membrane | 2.42E-04 | 1.28E-01 | [7](https://toppgene.cchmc.org/showQueryTerms.jsp?userdata_id=f345aaf3-1290-4655-affc-702083b2cfb3&feature=goc&row=6) | [55](https://toppgene.cchmc.org/showTermDetail.jsp?userdata_id=f345aaf3-1290-4655-affc-702083b2cfb3&category=GeneOntologyCellularComponent&id=GO:0012507) |
| 40 | GO:0030670 | phagocytic vesicle membrane | 4.18E-04 | 2.22E-01 | [7](https://toppgene.cchmc.org/showQueryTerms.jsp?userdata_id=f345aaf3-1290-4655-affc-702083b2cfb3&feature=goc&row=7) | [60](https://toppgene.cchmc.org/showTermDetail.jsp?userdata_id=f345aaf3-1290-4655-affc-702083b2cfb3&category=GeneOntologyCellularComponent&id=GO:0030670) |

**Supplemental Table 3: Summary information of the pathway analysis**

|  | ID | Name | Source | p-value | Bonferroni-corrected p-value | Genes from input | Genes in Annotation |
| --- | --- | --- | --- | --- | --- | --- | --- |
| 1 | P00012 | Cadherin signaling pathway | [PantherDB](https://toppgene.cchmc.org/output.jsp?userdata_id=f345aaf3-1290-4655-affc-702083b2cfb3) | 2.62E-10 | 3.37E-07 | [21](https://toppgene.cchmc.org/showQueryTerms.jsp?userdata_id=f345aaf3-1290-4655-affc-702083b2cfb3&feature=pt&row=0) | [159](https://toppgene.cchmc.org/showTermDetail.jsp?userdata_id=f345aaf3-1290-4655-affc-702083b2cfb3&category=Pathway&id=P00012) |
| 2 | 1269194 | Antigen Presentation: Folding, assembly and peptide loading of class I MHC | [BioSystems: REACTOME](http://www.ncbi.nlm.nih.gov/biosystems/1269194) | 1.56E-06 | 2.00E-03 | [7](https://toppgene.cchmc.org/showQueryTerms.jsp?userdata_id=f345aaf3-1290-4655-affc-702083b2cfb3&feature=pt&row=1) | [25](https://toppgene.cchmc.org/showTermDetail.jsp?userdata_id=f345aaf3-1290-4655-affc-702083b2cfb3&category=Pathway&id=1269194) |
| 3 | P00057 | Wnt signaling pathway | [PantherDB](https://toppgene.cchmc.org/output.jsp?userdata_id=f345aaf3-1290-4655-affc-702083b2cfb3) | 1.56E-06 | 2.01E-03 | [23](https://toppgene.cchmc.org/showQueryTerms.jsp?userdata_id=f345aaf3-1290-4655-affc-702083b2cfb3&feature=pt&row=2) | [305](https://toppgene.cchmc.org/showTermDetail.jsp?userdata_id=f345aaf3-1290-4655-affc-702083b2cfb3&category=Pathway&id=P00057) |
| 4 | 1269198 | Endosomal/Vacuolar pathway | [BioSystems: REACTOME](http://www.ncbi.nlm.nih.gov/biosystems/1269198) | 5.69E-06 | 7.32E-03 | [5](https://toppgene.cchmc.org/showQueryTerms.jsp?userdata_id=f345aaf3-1290-4655-affc-702083b2cfb3&feature=pt&row=3) | [12](https://toppgene.cchmc.org/showTermDetail.jsp?userdata_id=f345aaf3-1290-4655-affc-702083b2cfb3&category=Pathway&id=1269198) |
| 5 | 1470922 | Butyrophilin (BTN) family interactions | [BioSystems: REACTOME](http://www.ncbi.nlm.nih.gov/biosystems/1470922) | 5.69E-06 | 7.32E-03 | [5](https://toppgene.cchmc.org/showQueryTerms.jsp?userdata_id=f345aaf3-1290-4655-affc-702083b2cfb3&feature=pt&row=4) | [12](https://toppgene.cchmc.org/showTermDetail.jsp?userdata_id=f345aaf3-1290-4655-affc-702083b2cfb3&category=Pathway&id=1470922) |
| 6 | 83074 | Antigen processing and presentation | [BioSystems: KEGG](http://www.ncbi.nlm.nih.gov/biosystems/83074) | 1.63E-05 | 2.10E-02 | [10](https://toppgene.cchmc.org/showQueryTerms.jsp?userdata_id=f345aaf3-1290-4655-affc-702083b2cfb3&feature=pt&row=5) | [77](https://toppgene.cchmc.org/showTermDetail.jsp?userdata_id=f345aaf3-1290-4655-affc-702083b2cfb3&category=Pathway&id=83074) |
| 7 | 1269314 | Interferon gamma signaling | [BioSystems: REACTOME](http://www.ncbi.nlm.nih.gov/biosystems/1269314) | 1.70E-05 | 2.18E-02 | [11](https://toppgene.cchmc.org/showQueryTerms.jsp?userdata_id=f345aaf3-1290-4655-affc-702083b2cfb3&feature=pt&row=6) | [94](https://toppgene.cchmc.org/showTermDetail.jsp?userdata_id=f345aaf3-1290-4655-affc-702083b2cfb3&category=Pathway&id=1269314) |
| 8 | 83123 | Allograft rejection | [BioSystems: KEGG](http://www.ncbi.nlm.nih.gov/biosystems/83123) | 3.12E-05 | 4.01E-02 | [7](https://toppgene.cchmc.org/showQueryTerms.jsp?userdata_id=f345aaf3-1290-4655-affc-702083b2cfb3&feature=pt&row=7) | [38](https://toppgene.cchmc.org/showTermDetail.jsp?userdata_id=f345aaf3-1290-4655-affc-702083b2cfb3&category=Pathway&id=83123) |
| 9 | 1269192 | Class I MHC mediated antigen processing & presentation | [BioSystems: REACTOME](http://www.ncbi.nlm.nih.gov/biosystems/1269192) | 4.75E-05 | 6.11E-02 | [23](https://toppgene.cchmc.org/showQueryTerms.jsp?userdata_id=f345aaf3-1290-4655-affc-702083b2cfb3&feature=pt&row=8) | [376](https://toppgene.cchmc.org/showTermDetail.jsp?userdata_id=f345aaf3-1290-4655-affc-702083b2cfb3&category=Pathway&id=1269192) |
| 10 | 1269197 | ER-Phagosome pathway | [BioSystems: REACTOME](http://www.ncbi.nlm.nih.gov/biosystems/1269197) | 4.80E-05 | 6.17E-02 | [10](https://toppgene.cchmc.org/showQueryTerms.jsp?userdata_id=f345aaf3-1290-4655-affc-702083b2cfb3&feature=pt&row=9) | [87](https://toppgene.cchmc.org/showTermDetail.jsp?userdata_id=f345aaf3-1290-4655-affc-702083b2cfb3&category=Pathway&id=1269197) |
| 11 | 83124 | Graft-versus-host disease | [BioSystems: KEGG](http://www.ncbi.nlm.nih.gov/biosystems/83124) | 5.21E-05 | 6.71E-02 | [7](https://toppgene.cchmc.org/showQueryTerms.jsp?userdata_id=f345aaf3-1290-4655-affc-702083b2cfb3&feature=pt&row=10) | [41](https://toppgene.cchmc.org/showTermDetail.jsp?userdata_id=f345aaf3-1290-4655-affc-702083b2cfb3&category=Pathway&id=83124) |
| 12 | 83095 | Type I diabetes mellitus | [BioSystems: KEGG](http://www.ncbi.nlm.nih.gov/biosystems/83095) | 7.17E-05 | 9.22E-02 | [7](https://toppgene.cchmc.org/showQueryTerms.jsp?userdata_id=f345aaf3-1290-4655-affc-702083b2cfb3&feature=pt&row=11) | [43](https://toppgene.cchmc.org/showTermDetail.jsp?userdata_id=f345aaf3-1290-4655-affc-702083b2cfb3&category=Pathway&id=83095) |
| 13 | 1269195 | Antigen processing-Cross presentation | [BioSystems: REACTOME](http://www.ncbi.nlm.nih.gov/biosystems/1269195) | 2.01E-04 | 2.59E-01 | [10](https://toppgene.cchmc.org/showQueryTerms.jsp?userdata_id=f345aaf3-1290-4655-affc-702083b2cfb3&feature=pt&row=12) | [103](https://toppgene.cchmc.org/showTermDetail.jsp?userdata_id=f345aaf3-1290-4655-affc-702083b2cfb3&category=Pathway&id=1269195) |
| 14 | 1269171 | Adaptive Immune System | [BioSystems: REACTOME](http://www.ncbi.nlm.nih.gov/biosystems/1269171) | 2.30E-04 | 2.96E-01 | [37](https://toppgene.cchmc.org/showQueryTerms.jsp?userdata_id=f345aaf3-1290-4655-affc-702083b2cfb3&feature=pt&row=13) | [826](https://toppgene.cchmc.org/showTermDetail.jsp?userdata_id=f345aaf3-1290-4655-affc-702083b2cfb3&category=Pathway&id=1269171) |
| 15 | 1269609 | Negative regulation of TCF-dependent signaling by DVL-interacting proteins | [BioSystems: REACTOME](http://www.ncbi.nlm.nih.gov/biosystems/1269609) | 2.70E-04 | 3.48E-01 | [3](https://toppgene.cchmc.org/showQueryTerms.jsp?userdata_id=f345aaf3-1290-4655-affc-702083b2cfb3&feature=pt&row=14) | [6](https://toppgene.cchmc.org/showTermDetail.jsp?userdata_id=f345aaf3-1290-4655-affc-702083b2cfb3&category=Pathway&id=1269609) |
| 16 | 83121 | Autoimmune thyroid disease | [BioSystems: KEGG](http://www.ncbi.nlm.nih.gov/biosystems/83121) | 2.79E-04 | 3.58E-01 | [7](https://toppgene.cchmc.org/showQueryTerms.jsp?userdata_id=f345aaf3-1290-4655-affc-702083b2cfb3&feature=pt&row=15) | [53](https://toppgene.cchmc.org/showTermDetail.jsp?userdata_id=f345aaf3-1290-4655-affc-702083b2cfb3&category=Pathway&id=83121) |
| 17 | 125138 | Viral myocarditis | [BioSystems: KEGG](http://www.ncbi.nlm.nih.gov/biosystems/125138) | 5.44E-04 | 7.00E-01 | [7](https://toppgene.cchmc.org/showQueryTerms.jsp?userdata_id=f345aaf3-1290-4655-affc-702083b2cfb3&feature=pt&row=16) | [59](https://toppgene.cchmc.org/showTermDetail.jsp?userdata_id=f345aaf3-1290-4655-affc-702083b2cfb3&category=Pathway&id=125138) |

**Supplemental Table 4: List of AUC value of SVM method with different feature selection methods**

| No. of SNPs | Logistic (P<0.01) | | Logistic (P<0.05) | | LASSP (λ= 10^-3^) | | LASSP (λ= 10^-5^) | | LASSP (λ= 10^-7^) | | Logistic (P<0.01) & LASSP (λ= 10^-3^) | |
| --- | --- | --- | --- | --- | --- | --- | --- | --- | --- | --- | --- | --- |
|  | Test | Independent test set | Test | Independent test set | Test | Independent test set | Test | Independent test set | Test | Independent test set | Test | Independent test set |
| 10 | 0.552 | 0.563 | 0.486 | 0.526 | 0.502 | 0.503 | 0.529 | 0.510 | 0.497 | 0.510 | 0.586 | 0.608 |
| 100 | 0.686 | 0.648 | 0.613 | 0.580 | 0.611 | 0.550 | 0.613 | 0.534 | 0.553 | 0.493 | 0.684 | 0.764 |
| 500 | 0.721 | 0.720 | 0.723 | 0.695 | 0.773 | 0.546 | 0.716 | 0.541 | 0.547 | 0.502 | 0.776 | 0.897 |
| 1,000 | 0.742 | 0.738 | 0.758 | 0.740 | 0.877 | 0.517 | 0.770 | 0.510 | 0.564 | 0.505 | 0.812 | 0.911 |
| 1,500 | 0.764 | 0.755 | 0.778 | 0.757 | 0.946 | 0.475 | 0.832 | 0.538 | 0.599 | 0.535 | NA | NA |
| 2,000 | 0.785 | 0.764 | 0.797 | 0.777 | 0.972 | 0.486 | 0.875 | 0.472 | 0.600 | 0.526 | NA | NA |

**Supplemental Table 5: List of AUC value of RF method with different feature selection methods**

| No. of SNPs | Logistic (P<0.01) | | Logistic (P<0.05) | | LASSP (λ= 10^-3^) | | LASSP (λ= 10^-5^) | | LASSP (λ= 10^-7^) | | Logistic (P<0.01) & LASSP (λ= 10^-3^) | |
| --- | --- | --- | --- | --- | --- | --- | --- | --- | --- | --- | --- | --- |
|  | Test | Independent test set | Test | Independent test set | Test | Independent test set | Test | Independent test set | Test | Independent test set | Test | Independent test set |
| 10 | 0.528 | 0.549 | 0.538 | 0.534 | 0.556 | 0.585 | 0.527 | 0.589 | 0.534 | 0.484 | 0.540 | 0.539 |
| 100 | 0.636 | 0.601 | 0.566 | 0.550 | 0.610 | 0.542 | 0.576 | 0.508 | 0.559 | 0.512 | 0.637 | 0.632 |
| 500 | 0.665 | 0.667 | 0.620 | 0.584 | 0.681 | 0.491 | 0.627 | 0.495 | 0.582 | 0.504 | 0.702 | 0.682 |
| 1,000 | 0.681 | 0.673 | 0.641 | 0.618 | 0.724 | 0.518 | 0.632 | 0.507 | 0.584 | 0.493 | 0.696 | 0.723 |
| 1,500 | 0.708 | 0.669 | 0.656 | 0.619 | 0.723 | 0.520 | 0.660 | 0.523 | 0.564 | 0.513 | NA | NA |
| 2,000 | 0.708 | 0.677 | 0.658 | 0.629 | 0.739 | 0.548 | 0.668 | 0.502 | 0.591 | 0.549 | NA | NA |


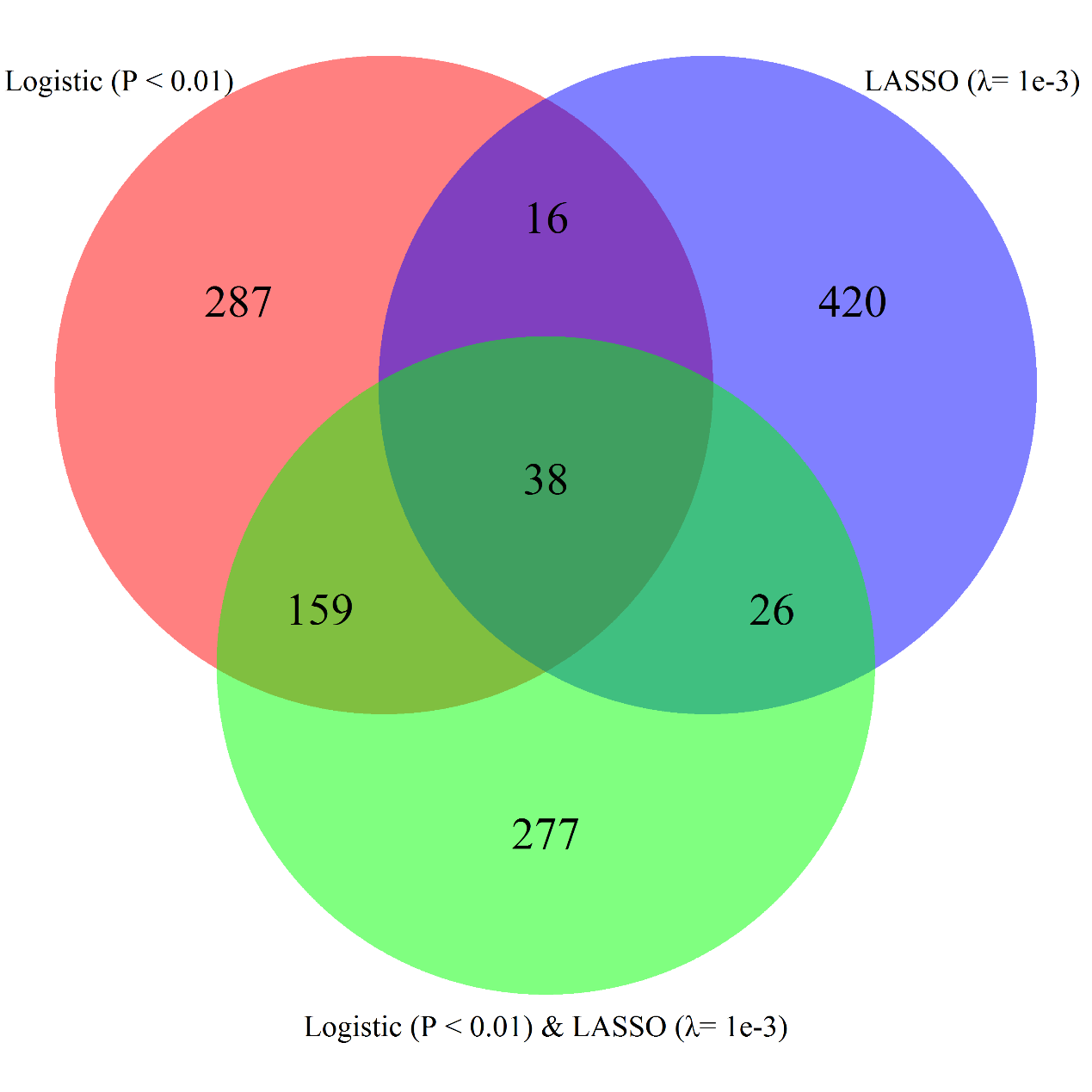


**Supplemental Figure 1: A comparison of top 500 SNPs selected by logistic regression at a P-value of <0.01, LASSO regression with a λ=10^-3^ , and the combined approach of logistic and LASSO regression**
